# Induction of filopodia formation by α-Actinin-2 via RelA with a feedforward activation loop promoting overt bone marrow metastasis of gastric cancer

**DOI:** 10.1186/s12967-023-04156-w

**Published:** 2023-06-19

**Authors:** Caiqin Wang, Bo Xie, Shi Yin, Jianghua Cao, Junhao Huang, Longyang Jin, Ge Du, Xiaohui Zhai, Rongqin Zhang, Shanshan Li, Taiyuan Cao, Hongen Yu, Xinjuan Fan, Zuli Yang, Junsheng Peng, Jian Xiao, Lei Lian

**Affiliations:** 1 Department of Gastrointestinal Surgery and Department of Medical Oncology, the Sixth Affiliated Hospital, Sun Yat-Sen University, Guangzhou, 510655 China; 2 Department of Forensic Toxicology, Faculty of Forensic Medicine, Zhongshan School of Medicine, Sun Yat-Sen University, Guangzhou, 510089 China; 3 State Key Laboratory of Oncology in South China, Collaborative Innovation Center for Cancer Medicine, Guangdong Key Laboratory of Nasopharyngeal Carcinoma Diagnosis and Therapy, Sun Yat-Sen University Cancer Center, Guangzhou, 510060 China; 4 Department of Nuclear Medicine, Guangdong Provincial Key Laboratory of Colorectal and Pelvic Floor Diseases, the Sixth Affiliated Hospital, Sun Yat-Sen University, Guangzhou, 510655 China; 5 Department of Pathology, Guangdong Provincial Key Laboratory of Colorectal and Pelvic Floor Diseases, the Sixth Affiliated Hospital, Sun Yat-Sen University, Guangzhou, 510655 China; 6 Guangdong Institute of Gastroenterology, Guangdong Provincial Key Laboratory of Colorectal and Pelvic Floor Diseases, the Sixth Affiliated Hospital, Sun Yat-Sen University, Guangzhou, 510655 China; 7 Department of General Surgery, the Sixth Affiliated Hospital, Sun Yat-Sen University, Guangzhou, 510655 China

**Keywords:** Gastric cancer, Bone marrow metastasis, α-Actinin-2, Filopodia formation, RelA

## Abstract

**Background:**

Bone marrow metastasis (BMM) is underestimated in gastric cancer (GC). GC with BMM frequently complicate critical hematological abnormalities like diffused intravascular coagulation and microangiopathic hemolytic anemia, which constitute a highly aggressive GC (HAGC) subtype. HAGC present a very poor prognosis with peculiar clinical and pathological features when compared with not otherwise specified advanced GC (NAGC). But the molecular mechanisms underlying BMM from GC remain rudimentary.

**Methods:**

The transcriptomic difference between HAGC and NAGC were analyzed. Genes that were specifically upregulated in HAGC were identified, and their effect on cell migration and invasion was studied. The function of ACTN2 gene were confirmed by GC cell lines, bone-metastatic animal model and patients’ tissues. Furthermore, the molecular mechanism of ACTN2 derived-BMM was explored by multiple immunofluorescence staining, western blot, chromatin immunoprecipitation, and luciferase reporter assays.

**Results:**

We elucidated the key mechanisms of BMM depending on the transcriptomic difference between HAGC and NAGC. Five genes specifically upregulated in HAGC were assessed their effect on cell migration and invasion. The ACTN2 gene encoding protein α-Actinin-2 was detected enhanced the metastatic capability and induced BMM of GC cells in mouse models. Mechanically, α-Actinin-2 was involved in filopodia formation where it promoted the Actin filament cross-linking by replacing α-Actinin-1 to form α-Actinin-2:α-Actinin-4 complexes in GC cells. Moreover, NF-κB subunit RelA and α-Actinin-2 formed heterotrimers in the nuclei of GC cells. As a direct target of RelA:α-Actinin-2 heterotrimers, the ACTN2 gene was a positive auto-regulatory loop for α-Actinin-2 expression.

**Conclusions:**

We demonstrated a link between filopodia, BMM and ACTN2 activation, where a feedforward activation loop between ACTN2 and RelA is established via actin in response to distant metastasis. Given the novel filopodia formation function and the new mechanism of BMM in GC, we propose ACTN2 as a druggable molecular vulnerability that may provide potential therapeutic benefit against BMM of GC.

**Supplementary Information:**

The online version contains supplementary material available at 10.1186/s12967-023-04156-w.

## Introduction

Liver, peritoneum and lung are the most commonly metastatic sites of gastric cancer (GC) [1]. Cancer cells have also been found in bone marrow (BM) up to 20% of GC through autopsy [2], which indicates BM metastasis (BMM) might be underestimated in advanced GC (AGC). BMM has been used referring to a broad spectrum of BM infiltration varying from micro-metastasis to overt disseminated carcinomatosis [3]. AGC with overt BMM frequently complicate critical hematological disorders like disseminated intravascular coagulation (DIC), which present as aggressive disease entity. We firstly defined it as highly aggressive GC (HAGC) with its peculiar clinicopathological features [4]. Compare to non-specific AGC (NAGC), HAGC was characterized with the double occurrence of multiple BMM and DIC. In additional, HAGC was reported with hyperinflammation in our previous study [4]. Therefore, HAGC had highly aggressive biological behavior and very poor prognosis. The prospective phase II trial has also been initiated [5]. While the molecular mechanism underlying BMM is little known.

The α-Actinin-2 (encoded by *ACTN2* gene) is a member of the α-Actinin family of F-Actin crosslinking proteins [6]. Four human α-Actinin members have been described: α-Actinin-1, 2, 3 and 4 [6]. α-Actinin-1 and α-Actinin-4 are ubiquitously expressed and have major functions in cytoskeleton regulation [6]. α-Actinin-2 is mainly expressed in the myocardium, skeletal muscle and brain [7], while α-Actinin-3 appears mostly in the skeletal muscle [6]. α-Actinin-2 includes three functional domains: the NH2-terminal region that mediates interactions with the Actin rod, the central region composed of four spectrin-like repeats, and the COOH-terminal that contains an EF-hand [8]. In humans, mutations in the *ACTN2* gene cause dilated cardiomyopathy [9]; however, the exact function of α-Actinin-2 in the development of cancer is largely unknown.

Filopodia are thin (0.1–0.3 μm in diameter) finger-like, Actin-rich plasma membrane protrusions that are involved in numerous cellular processes, including cancer cell migration and neuronal growth-cone pathfinding [10]. Abundant filopodia is a hallmark of tumor cells and has been observed for many invasive cancer cell types [10]. Interestingly, spines on neuronal dendrites represent a filopodium-like protrusion morphology, and α-Actinin-2 plays a key part in increasing the length and density of dendritic protrusions on hippocampal neurons [11].

Here, we report a novel filopodia formation function of α-Actinin-2 that was shown to have oncogenic roles in HAGC and shown correlation with poor prognosis. The resulting positive auto-regulatory loop of the *ACTN2* gene involving the transcriptional activation complex NF-κB subunit p65 (RelA): α-Actinin-2 provides new insight into the mechanism of HAGC pathogenesis and progress.

## Results

### *ACTN2* is overexpressed in GC patients with BMM

**Fig. 1:**
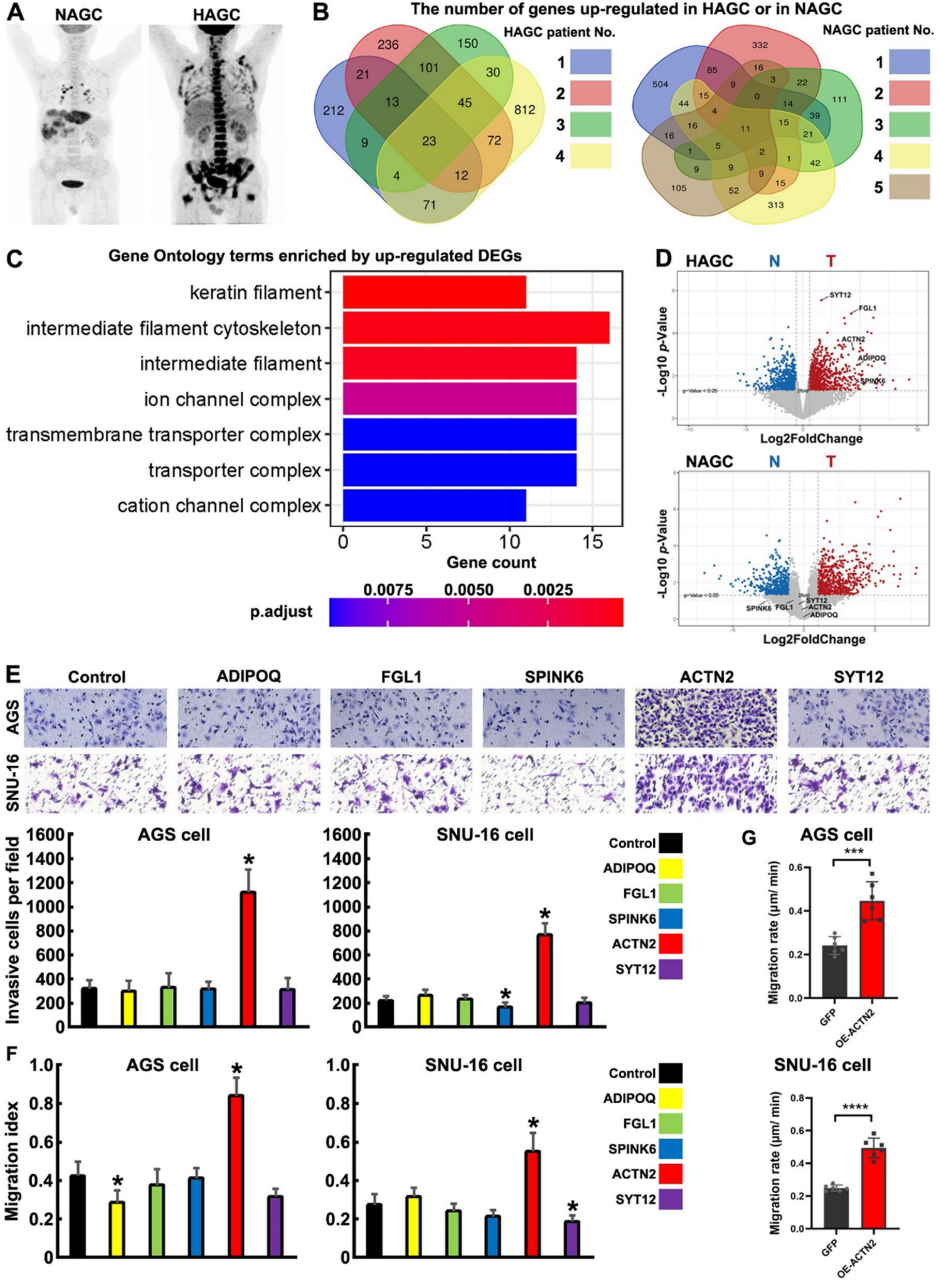
*ACTN2* is a gene associated with BMM in patients with GC. **A** Whole-body maximum-intensity projection PET image demonstrates the location and high ^18^F-fluorodeoxyglucose activity of the tumor in NAGC patient and HAGC patient. **B** 23 genes overexpressed between adjacent normal tissues and tumor samples of HAGC, 11 genes overexpressed between adjacent normal tissues and tumor samples of NAGC. The label in the upper right corner of the figure, each different color represents a different patient. There were four patients in the HAGC group and five patients in the NAGC group. **C** The enriched (count ≥ 5) gene ontology terms of up-regulated differentially expressed genes (DEGs) of HAGC patients compared to NAGC patients. **D** *ADIPOQ*, *FGL1*, *SPINK6*, *ACTN2* and *SYT12* are specifically upregulated in HAGC but not in NAGC or in The Human Protein Atlas dataset. **E** and **F** GC cells invasion and migration after 24 h *ADIPOQ*, *FGL1*, *SPINK6*, *ACTN2* and *SYT12* transfection were examined by **E** transwell chamber assays or **F** wound healing assays. The number of invading tumor cells was counted using 5 high-intensity fields. G 2D single cell migration assessment of GFP and OE-ACTN2 cells (scale bar 10 μm). The bars indicate the SD. The results are expressed as the mean ± SD of five independent experiments. *P < 0.05, ***P < 0.001, ****P < 0.0001, using Student’s *t*-test

Through retrospective analysis, we screened a series of fluorodeoxyglucose‑positron emission tomography/CT (FDG‑PET/CT) imagesfrom patients with HAGC or NAGC. We found HAGCwere complicated with diffuse BMM (Fig. 1A). The transcriptomes of these HAGC patients and NAGC patients without BMM were profiled by RNA-seq. Detailed patient characteristics and clinical-pathological parameters are shown in Additional file 1: Table S1. In HAGC, a threshold FDR < 0.05 identified 23 genes that were significantly overexpressed and no downregulated gene between adjacent normal tissues and tumor samples. In NAGC, a threshold FDR < 0.05 identified in 11 genes significantly overexpressed between adjacent normal tissues and tumor samples (Fig. 1B and Additional file 1: Table S2). To identify the specific functional processes associated with BMM, we performed Gene Ontology (GO) analysis to visualize 16 differentially expressed genes (DEGs) of HAGC patients compared to NAGC patients (Additional file 1: Table S2). GO terms enrichment analysis indicated that the up-regulated DEGs of HAGC were mainly enriched in keratin filament, intermediate filament cytoskeleton and intermediate filament (Fig. 1C). In terms of molecular function, up-regulated DEGs of HAGC were significantly enriched in structural molecule activity and cell dynamic structures, suggesting that the abnormal expression of cytoskeletal and cytoskeletal-associated proteins play an important role in the ability of cancer cells to BMM.

In order to find the specific molecules that regulate BMM of GC, the 16 DEGs of HAGC were further excluded by comparison with the data of GC patients without BMM from The Human Protein Atlas (https://www.proteinatlas.org/). With a threshold of average FPKM < 1, *ADIPOQ* (encoding a protein called Adiponectin, which is a homeostatic factor for regulating glucose levels, lipid metabolism, and insulin sensitivity), *SPINK6* (encoding a protein called Serine peptidase inhibitor Kazal type 6, which is a Kazal-type serine protease inhibitor that acts on kallikrein-related peptidases in the skin), *ACTN2* (encoding an Actin-binding protein called α-Actinin-2) and *SYT12* (encoding an protein called Synaptotagmin 12 that mediates calcium-dependent regulation of membrane trafficking in synaptic transmission) were specifically upregulated in HAGC but were not detected in the data of GC patients without BMM from The Human Protein Atlas (Fig. 1D and Additional file 1: Table S2).

Considering the obvious difference between HAGC and NAGC, where HAGC cells have a strong ability to undergo distant metastasis, we focused on the effects of *ADIPOQ*, *SPINK6*, *ACTN2*, *SYT12* and *FGL1* (since the Human Protein Atlas do not show the protein level of *FGL1*, we included it in this test) on promoting cell migration and invasion. These five genes were overexpressed in GC cell lines (AGS as GC in situ; SNU-16 as GC in ascites). In both transwell assay and wound healing assay, overexpression of *ACTN2* increased cell motility while*FGL1*and *SYT12* had no effect on GC cell motility. Moreover, *SPINK6* showed inhibition invasion of SNU-16 and *ADIPOQ* showed inhibition of migration of AGS (Fig. 1E and F). When co-expression of *ACTN2* and *ADIPOQ* in AGS, as well as co-expression of *ACTN2* and *SYT12*/*SPINK6* in SNU-16 cells, there were no weakness of the ability of *ACTN2* to promote cell migration cell invasion (Additional file 1: Fig. S1A–C). Furthermore, Single cell migration assay was good at detecting the cell migration. Through single cell migration assay [12], α-actinin-2 overexpressed AGS and SNU-16 cells showed significantly higher rate of migration (Fig. 1G). These results suggested that *ACTN2* may be a key regulator in GC cell metastasis.

### α-Actinin-2 promotes GC cell migration by facilitating the increase and growth of filopodia

**Fig. 2:**
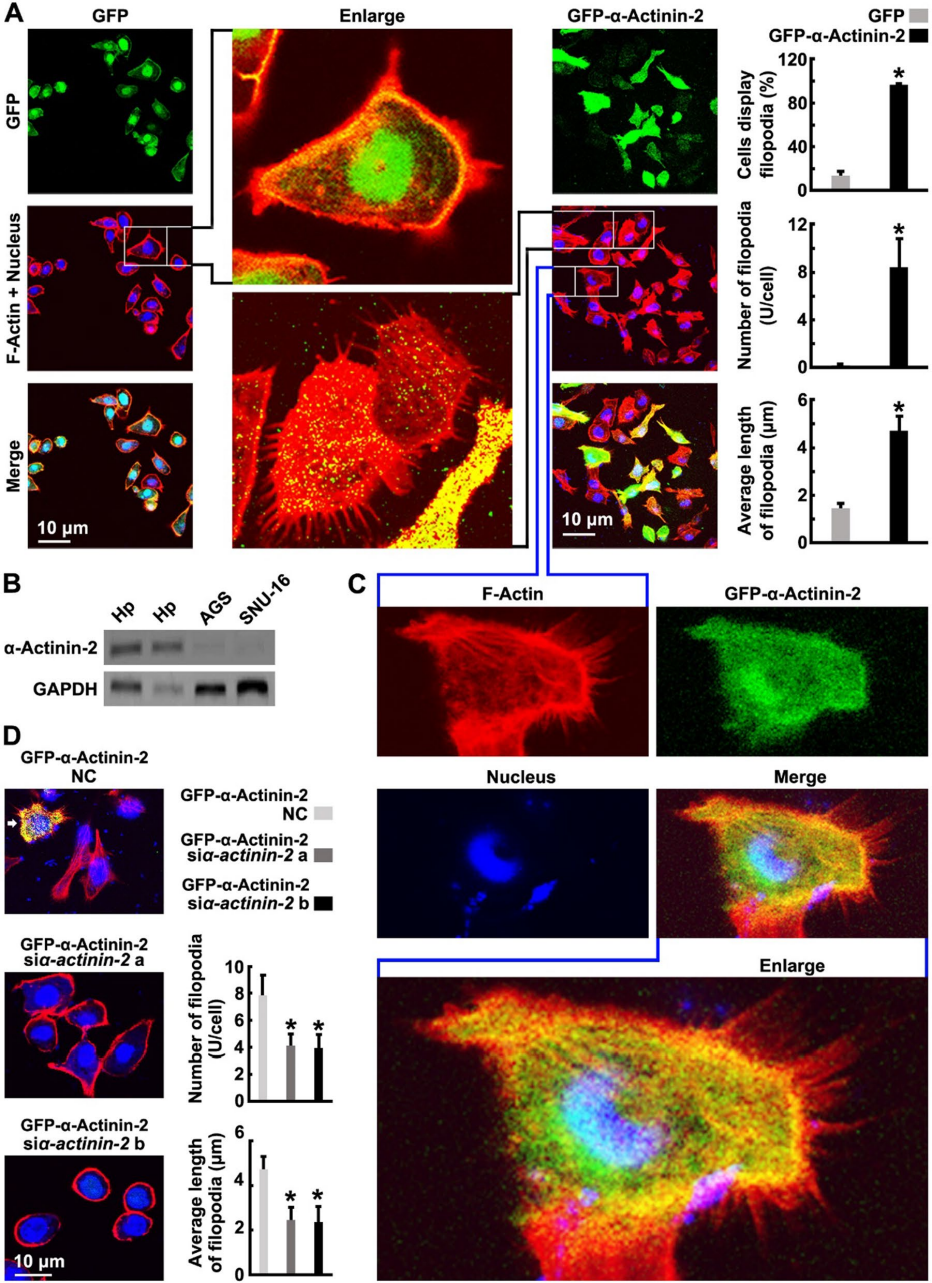
α-Actinin-2 promotes GC cell migration by facilitating the increase and growth of filopodia. **A** and **C** AGS cells were transfected with GFP or GFP-α-Actinin-2 for 24 h, then cells were stained for quantification of the percentage of cells display filopodia, the number of filopodia per cell or the average length of filopodia. **B** Tissue and cell lysates were harvested and subjected to Western blotting using indicated antibodies, the sampling amount of the second band is one-third of that of the first band. **D** AGS cells were transfected with GFP-α-Actinin-2 for 2 days following transfect with negative control (NC) siRNAs or α-Actinin-2 siRNAs for 24 h, then cells were stained for quantification of the number of filopodia per cell or the average length of filopodia. The bars indicate the SD. The results are expressed as the mean ± SD of three independent experiments. *P < 0.05 using Student’s *t*-test for (**A**). *P < 0.05 using Ordinary one-way ANOVA for (**D**). GFP/GFP-α-Actinin-2 (green), F-Actin (red) and Nucleus (DAPI, blue). Scale bar, 10 μm

To assess whether the cytoskeleton is involved in α-Actinin-2-mediated cell migration, we labeled the main component of the cytoskeleton, fibrous Actin (F-Actin), with an F-Actin specific probe, TRITC-conjugated phalloidin (Fig. 2A). Endogenous α-Actinin-2 was found to be abundantly expressed in crude mouse hippocampus but undetectable in AGS, SNU-16 cells (Fig. 2B). F-Actin staining revealed overexpression of α-Actinin-2 significantly increased the number and length of filopodia in AGS cells (Fig. 2A) and SNU-16 cells (Additional file 1: Fig. S2A), without affect the viability and apoptosis of cells (Additional file 1: Fig. S2B–E). In AGS cells, only 13.46% of the cells had a few short filopodia (number: 0.199 U/cell; average length: 1.47 μm; Fig. 2A). In α-Actinin-2 overexpressed AGS cells, more than 96% cells displayed numerous thin and long filopodia (number: 8.41 U/cell; average length: 4.69 μm; Fig. 2A).

Overexpression of α-Actinin-2 revealed a whole-cell distribution of this protein, α-Actinin-2 was distributed in the nucleus, cytoplasm, membrane and filopodia (Fig. 2C). However, α-Actinin-2 was localized to the proximal part of filopodia and gradually decreased from the proximal portion to the distal portion (Fig. 2C). Knockdown of α-Actinin-2 with siRNA after 2 days of α-Actinin-2 overexpression inhibited the increase in filopodia. Analysis of three randomly chosen cell groups revealed that the number and length of filopodia were significantly decreased after α-Actinin-2 knockdown (Fig. 2D). These results indicated that α-Actinin-2 is required for the increase and growth of filopodia.

### α-Actinin-2 mediates the polymerization and selective aggregation of Actin at filopodia

**Fig. 3:**
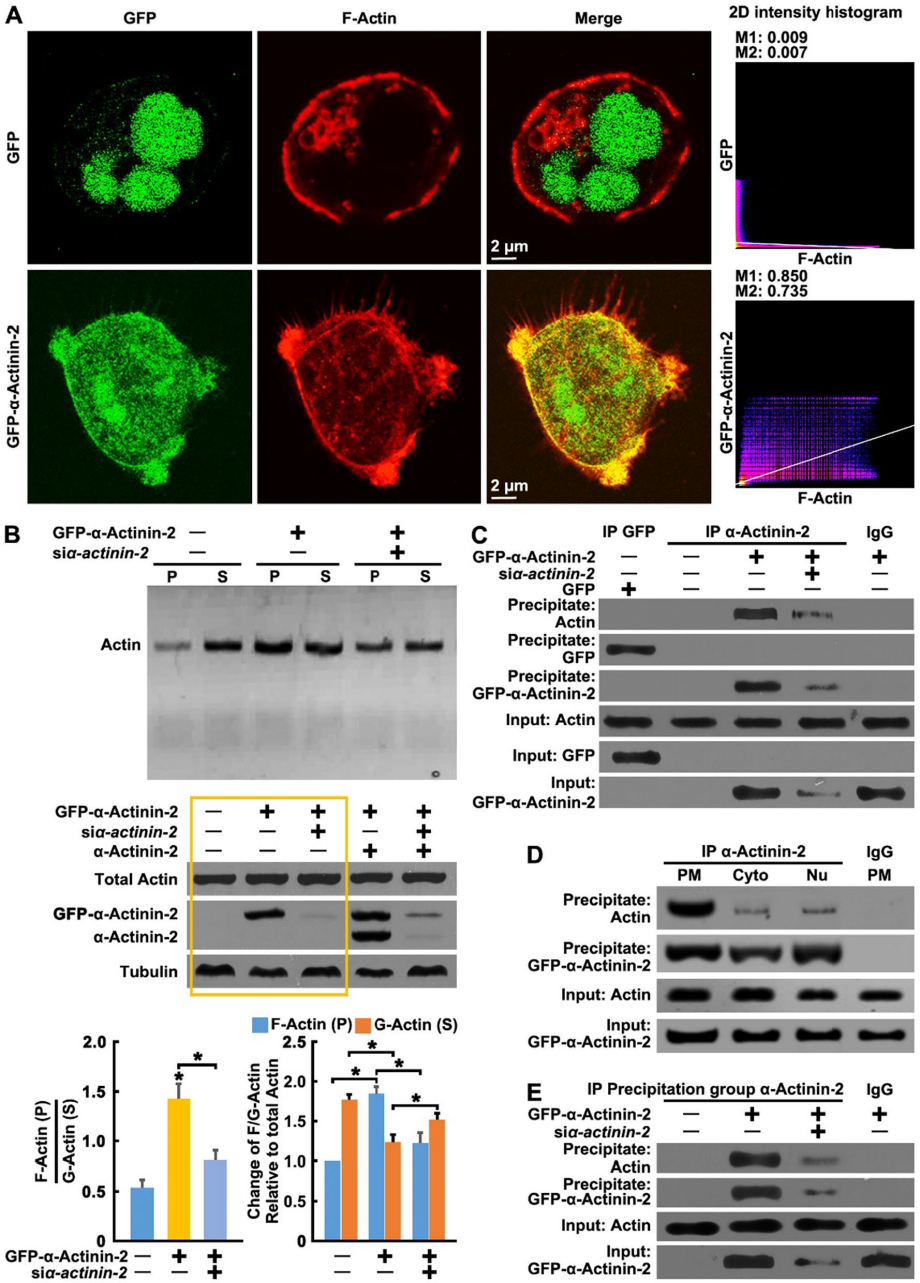
α-Actinin-2 mediates the polymerization and selective aggregation of Actin at filopodia. **A** AGS cells were transfected with GFP or GFP-α-Actinin-2 for 24 h, then the cells were stained for GFP/GFP-α-Actinin-2 (green) and F-Actin (red). The fluorescence colocalization of α-Actinin-2 and F-Actin was performed using the ImageJ Plugins software. Manders′ Coefficient 1 (M1): the percentage of GFP/GFP-α-Actinin-2 colocalized with F-Actin, Manders′ Coefficient 2 (M2): the percentage of F-Actin colocalized with GFP/GFP-α-Actinin-2. **B** GFP-α-Actinin-2 or α-Actinin-2 was co-transfected with or without α-Actinin-2 siRNAs in AGS cells for 24 h, then the ratio of F-Actin/G-Actin was determined by G-Actin and F-Actin ratio assays (upper and lower panel), the input of total Actin, Tubulin, GFP-α-Actinin-2 and α-Actinin-2 were also showed in the middle panel. P: pellet; S: supernatant. **C** GFP-α-Actinin-2 or GFP was co-transfected with or without α-Actinin-2 siRNAs in AGS cells for 24 h, then cells were lysed for co-Immunoprecipitation (IP) assay by using α-Actinin-2 antibodies. **D** AGS cells were transfected with α-Actinin-2 for 24 h, then the membrane extract (PM), cytoplasmic extract (Cyto) and nuclear extract (Nu) of cells were separated by the Subcellular Protein Fractionation Kit. Immunoprecipitation (IP) assays were performed in PM, Cyto and Nu by using α-Actinin-2 antibodies. **E** GFP-α-Actinin-2 was co-transfected with or without α-Actinin-2 siRNAs in AGS cells for 24 h, then cells were treated as in (**B**) to separate the supernatant and pellet. The supernatant was used for co-Immunoprecipitation (IP) assay by using α-Actinin-2 antibodies. The bars indicate the SD. The results are expressed as the mean ± SD of three independent experiments. *P < 0.05 using Ordinary one-way ANOVA

No significant colocalization of overexpressed α-Actinin-2 and phalloidin-labeled F-Actin in any part of the cell was observed, except at the cell-membrane and the proximal region of the filopodia (Fig. 3A). Interestingly, F-Actin was evenly distributed throughout the cell membrane of GC cells and was locally clustered in the proximal part of filopodia upon α-Actinin-2 overexpression (Fig. 3A).

F-Actin complexes are polymers assembled from globular Actin monomers (G-Actin). To confirm whether α-Actinin-2 contributes to the growth of filopodia through G-Actin assembly, we measured the ratio of the two forms of this protein. Cells were lysed in a detergent-based lysis buffer that stabilizes and maintains the G- and F- forms of cellular Actin. The buffer solubilizes G-actin but will not solubilize F-Actin. A centrifugation step pellets the F-Actin and leaves the G-Actin in the supernatant. Samples of supernatant and pellet are run in an SDS-PAGE system and actin is quantitated by western blot analysis. Overexpression of α-Actinin-2 was found to increase the ratio of F-Actin to G-Actin, while knock-down of α-Actinin-2 decreased this ratio (Fig. 3B). This finding is consistent with a recent study that showed that breast cancer cells with bone tropism are much stiffer with enhanced F-Actin [13].

Further, co-immunoprecipitation assay was used to analyze the interaction between α-Actinin-2 and Actin. However, many of the commercially available antibodies for α-Actinin-2 cross-react with α-Actinin isoforms. To clarify this issue, we used antibodies that is specific for α-Actinin 1,2,3 or 4 (the immunogen of these antibodies is indicated in Additional file 1: Fig. S3A and S3B) and does not cross-react between α-Actinin isoforms 1, 2, 3 and 4, which are present in AGS cells by overexpression GFP-α-Actinin 1,2,3 or 4 (Additional file 1: Fig. S3B). Using the specific antibody, the co-immunoprecipitation assay revealed that α-Actinin-2 and Actin form a stable complex mainly in the plasma membrane component of cell, but only a small amount α-Actinin-2/Actin complex formed in the cytoplasm and nucleus (Fig. 3C and D). Further, we found that α-Actinin-2 mainly interact with F-Actin (Fig. 3E) but not G-Actin (Additional file 1: Fig. S3D). These data suggest that α-Actinin-2 directly binds to Actin and may promotes its assembly into filopodia.

### α-Actinin-2 enhances F-Actin binding ability by replacing α-Actinin-1 to form α-Actinin-2:α-Actinin-4 complexes

**Fig. 4:**
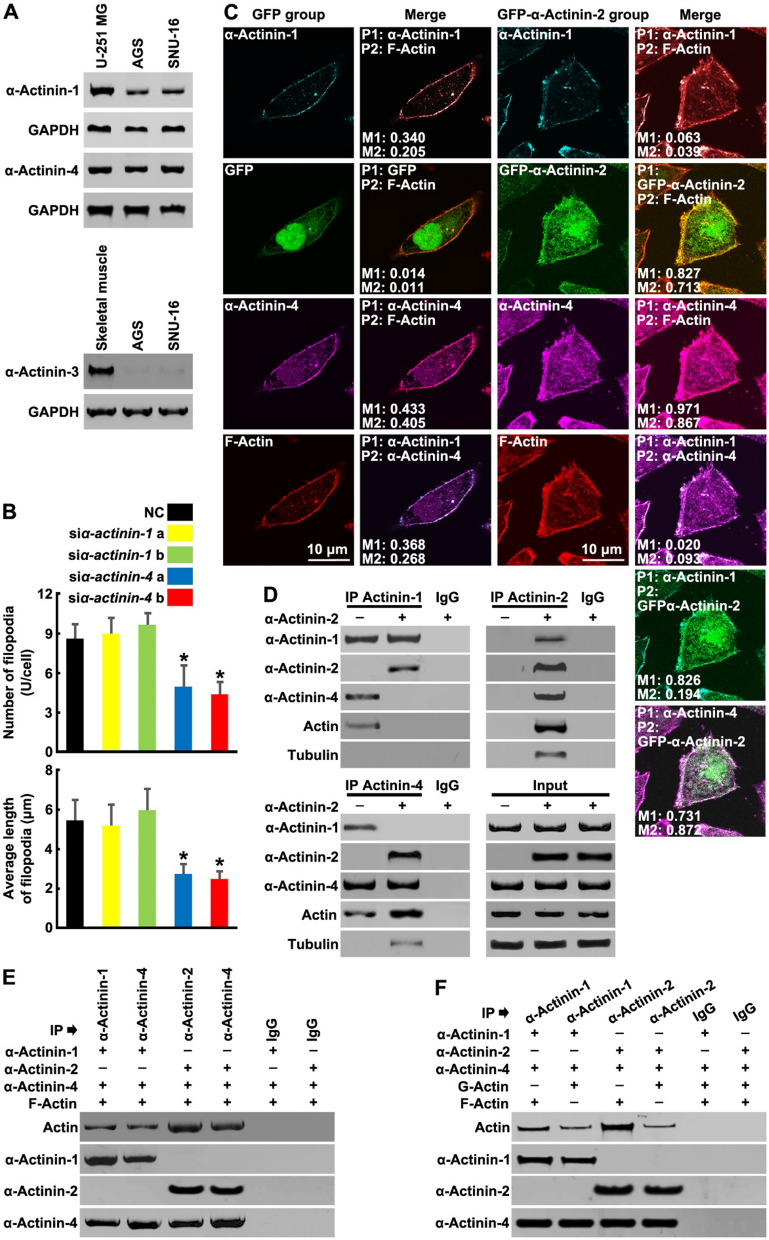
α-Actinin-2 enhances F-Actin binding ability by replacing α-Actinin-1 to form α-Actinin-2:α-Actinin-4 complexes. **A** Tissue and cell lysates were harvested and subjected to Western blotting using indicated antibodies. **B** AGS cells were transfected with GFP-α-Actinin-2 for 2 days following transfection with NC or α-Actinin-1/4 siRNAs for 24 h. The number of filopodia per cell and the average length of filopodia were quantified. The bars indicate the SD. The results are expressed as the mean ± SD of three independent experiments. *P < 0.05 using Ordinary one-way ANOVA. **C** AGS cells were transfected with GFP or GFP-α-Actinin-2 for 24 h, then the cells were stained for α-Actinin-1 (Cyan), GFP/GFP-α-Actinin-2 (green), α-Actinin-4 (magenta) and F-Actin (red), the fluorescence colocalization between α-Actinin-1/2/4 and F-Actin was performed using ImageJ Plugins software. Manders′ Coefficient 1 (M1): the percentage of P1 colocalized with P2, Manders′ Coefficient 2 (M2): the percentage of P2 colocalized with P1. **D** AGS cells were transfected with α-Actinin-2 for 24 h, then IP assays were performed using α-Actinin-1/2/4 antibodies. **E** and **F** In vitro G-Actin/F-Actin and α-Actinin-1:α-Actinin-4 complex or α-Actinin-2:α-Actinin-4 complex binding studies were performed and analyzed by Western blotting using the indicated antibodies

Multiple isoforms of α-Actinin are found in humans and are encoded by at least four distinct genes. We found that common GC cells contained two non-muscle α-Actinin isoforms, α-Actinin-1 and α-Actinin-4 (Fig. 4A). However, the skeletal muscle-specific α-Actinin isoforms, α-Actinin-2 and α-Actinin-3, were not detected (Figs. 2B and 4A). Interestingly, knockdown of α-Actinin-4, but not α-Actinin-1, with specific siRNAs after α-Actinin-2 overexpression inhibited the increase in the number and the length of filopodia (Fig. 4B), indicating that α-Actinin-4 is required for α-Actinin-2-induced filopodia formation.

Previous studies have shown that α-Actinins form anti-parallel dimers to function in various processes including invasion and migration of cancer cells [14, 15]. We observed that the colocalization between α-Actinin-1 and α-Actinin-4/F-Actin were decreased after α-Actinin-2 overexpression (Fig. 4C); α-Actinin-2 overexpression induced a significant colocalization of α-Actinin-2, α-Actinin-4 and F-Actin (Figs. 3A and 4C). Meanwhile, colocalization between α-Actinin-1 and α-Actinin-2 also occurred on the cell plasma membrane (Fig. 4C), but α-Actinin-1 depletion did not affect the complex formation between α-Actinin-4, α-Actinin-2 and Actin (Additional file 1: Fig. S4A).

Further, we tested the direct interaction between α-Actinins. α-Actinin-4 and a small amount Actin were detected following α-Actinin-1 pulldown, and the reverse was the same (Fig. 4D). Under the α-Actinin-2 overexpression condition, α-Actinin-2 was detected following α-Actinin-1 pulldown, but Actin and α-Actinin-4 were below the limits of detection; extensive α-Actinin-4, Actin and little α-Actinin-1 were detected following α-Actinin-2 pulldown; abundant α-Actinin-2 and Actin, but not α-Actinin-1, were detected following α-Actinin-4 pulldown (Fig. 4D). Therefore, upregulation of α-Actinin-2 resulted in a shift in α-Actinin dimer composition from α-Actinin-1:α-Actinin-4 to α-Actinin-2:α-Actinin-4 and α-Actinin-2:α-Actinin-1.

Next, we assessed whether α-Actinin-2:α-Actinin-4 complexes fulfill their basic role of binding to F-Actin using Actin co-sedimentation assays which are commonly used for identifying Actin-associated proteins[16]. Strong F-Actin binding was detected in α-Actinin-2:α-Actinin-4 complexes, in contrast to low F-Actin binding activity in α-Actinin-1:α-Actinin-4 complexes (Fig. 4E and inputs showed in Additional file 1: Fig. S4B). In addition, extensive binding of G-Actin was not detected in both α-Actinin-1:α-Actinin-4 and α-Actinin-2:α-Actinin-4 complexes (Fig. 4F and inputs showed in Additional file 1: Fig. S4C). Together, these results suggest α-Actinin-2 enhances F-Actin binding ability by replacing α-Actinin-1 to form α-Actinin-2:α-Actinin-4 complexes.

### *ACTN2* is a direct target gene of NF-κB signaling in GC cells

**Fig. 5:**
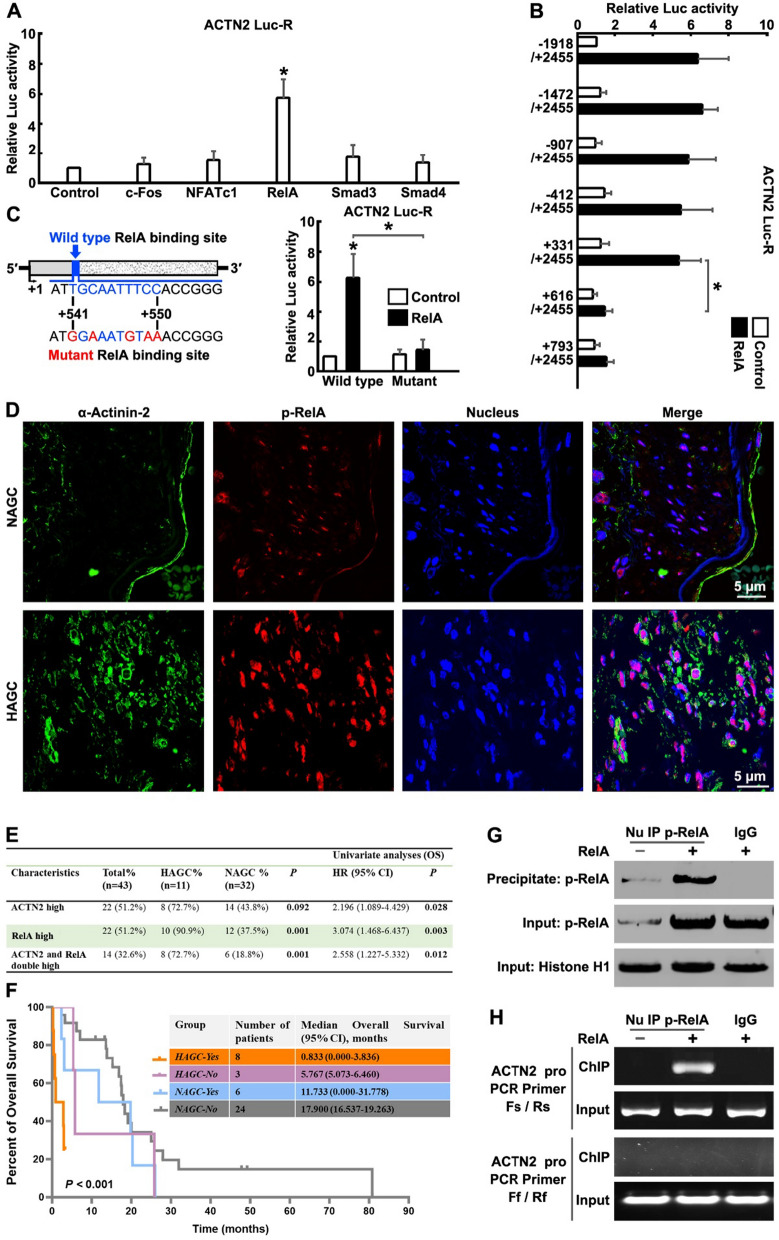
*ACTN2* is a direct target gene of the NF-κB signaling in GC cells. **A** c-Fos, NFATc1, RelA, Smad3 or Smad4 was cotransfacted with the *ACTN2* promoter (-1918/ + 2455) reporter gene (*ACTN2* Luc-R) into SNU-16 cells for 12 h and dual-luciferase reporter assays were performed. **B** *ACTN2* promoter 5′ sequential deletion constructs. Fragments of different lengths of the *ACTN2* promoter with the same 3′ end were cloned into pGL3-Basic. RelA was co-transfected with each of the *ACTN2* promoter-based reporters for 12 h and dual-luciferase reporter assays were performed. **C** Mutation of putative RelA binding site at the *ACTN2* promoter. SNU-16 cells were transfected with wild type or mutant *ACTN2* Luc-R for 12 h and dual-luciferase reporter assays were performed. The statistical analysis information in A-C: The bars indicate the SD. The results are expressed as the mean ± SD of three independent experiments. *P < 0.05 using Ordinary one-way ANOVA. **D** NAGC and HAGC tissue sections obtained after infusion were immunostained for α-Actinin-2 (green), Ser276 phosphorylated RelA (p-RelA, red) and nucleus (blue). **E** Differences in expression of α-Actinin-2 and p-RelA between HAGC and NAGC, and relationship of overall survival proportion of patients based on univariate analyses. *P < 0.05 using the two-sided Pearson chi-squared tests. **F** Overall survival proportion of patients with HAGC and NAGC stratified by α-Actinin-2 and p-RelA simultaneous high expression based on Kaplan–Meier survival analyses. HAGC-yes: α-Actinin-2 and p-RelA were simultaneously highly expressed in HAGC sample. HAGC-no: at least one marker in α-Actinin-2 and p-RelA had low expression in HAGC sample. Same to NAGC-yes and NAGC-no. *P < 0.05 using Kaplan–Meier plots and compared with the log-rank test. **G** and **H** SUN-16 cells were transfected with or without RelA for 24 h, then the cell nuclear extracts were subjected to ChIP analysis using the p-RelA antibody. **G** Western blotting analysis with the p-RelA antibody demonstrates the IP specificity and efficiency. **H** DNA isolated and purified from immunoprecipitated material was amplified with primers spanning the RelA binding site (Fs/Rs) or primers far away from the RelA binding site (Ff/Rf) of the *ACTN2* gene promoter

Previous studies have shown that the AKT/GSK3β/c-Fos/NFATc1 [17], IGF-1/AKT/NF-κB (RelA) [18] or TGF-β/Smad3/4 [19] signaling cascade play a key regulatory role in BMM of many cancers. To demonstrate which transcription factor may regulate the activation of the *ACTN2* gene promoter, c-Fos, NFATc1, RelA, Smad3 or Smad4 was cotransfected with *ACTN2* promoter (nucleotides from − 1918 to + 2455 relative to the transcription start site) reporter gene into GC cells. The results revealed RelA, but not c-Fos, NFATc1, Smad3 or Smad4, significantly activated the promoter activity of *ACTN2* (Fig. 5A), demonstrating an NF-κB-dependent manner of regulating α-Actinin-2 expression. Subsequently, we performed a pathway enrichment analysis on RNA-seq data of HAGC and NAGC. Following GO analysis, the pathway that exhibited specific enrichment in HAGC were NF-κB signaling pathway, positive regulation of NF-κB transcription factor activity, toll-like receptor signaling pathway and positive regulation of NIK/NF-κB signaling (Additional file 1: Fig. S5A–C). Notably, compared to NAGC, the genes with differential expression in HAGC are BCL2A1, BCL2L1, CCL13, CCL2, CCL3, CD14, CDK4, CDK6, IL6, MMP9, TNFA, et.al, all of which have been reported as transcriptional regulatory genes of RelA (Additional file 1: Fig. S5D) [20]. These results demonstrated that the NF-κB pathway was activated in HAGC.

Further, the core sequences required for the promoter activation were identified. A series of reporter constructs containing deletions of the 5′-flanking region of the *ACTN2* promoter were cloned into a reporter vector. All of the truncated *ACTN2* promoter constructs had the same 3′ end. One of the deletion constructs (nucleotides + 331 to + 2455) showed the same response as the original construct (nucleotides − 1918 to + 2455), whereas another deletion construct (nucleotides + 616 to + 2455) demonstrated almost completely abolished RelA-induced promoter activity (Fig. 5B). Thus, the principal *ACTN2* transcriptional control element was narrowed down to the region spanning nucleotides + 331 to + 616.

Promoter analysis identified a potential RelA binding site + 541/ + 550 (TGC AAT TTC C) in the core region of the Human *ACTN2* promoter, and mutation at this site showed clear disruption of *ACTN2* promoter activation in response to RelA treatment (Fig. 5C). Meanwhile, immunostaining was performed to detect the cellular co-localization of α-Actinin-2 and Ser276 phosphorylated RelA (p-RelA) in GC tissues. Compared with the NAGC group, the HAGC group exhibited significantly higher α-Actinin-2 and p-RelA expression, and α-Actinin-2 positive cells overlapped with p-RelA high expression cells, where the activated p-RelA subunit was localized in the nucleus and the α-Actinin-2 protein mainly in the membrane of the same cell (Fig. 5D and E). Detailed patient characteristics and clinical-pathological parameters are shown in Additional file 1: Table S4. Correlation analysis demonstrated high expression of any of α-Actinin-2 and p-RelA was associated with poor survival based on univariate analyses (Fig. 5E). Simultaneous high staining of α-Actinin-2 and p-RelA was significantly related to overall survival in HAGC but not in NAGC. HAGC cases with both high expression of α-Actinin-2 and p-RelA had the worst survival outcome (Fig. 5F). Overexpression of RelA has promoted the growth of filopodia (Additional file 1: Fig. S6A) and increase the ratio of F-Actin to G-Actin, while knock-down of α-Actinin-2 decreased this ratio (Additional file 1: Fig. S6B). These results suggested a close association between p-RelA and α-Actinin-2.

To confirm the direct interactions between RelA and the *ACTN2* promoter in vivo, a ChIP assay was used in GC cells in the presence or absence of RelA overexpression. Chromatin was precipitated with a p-RelA antibody. p-RelA protein that was cross-linked to DNA was readily detected in RelA-overexpressing cells (Fig. 5G). The precipitated DNA was assayed by PCR with primers spanning the RelA binding site + 541/ + 550 (Primer: Fs/Rs) or primers far from this site (Primer: Ff/Rf) on the *ACTN2* promoter. The DNA fragments from RelA-overexpressing cells were amplified strongly by PCR primers Fs/Rs but not by PCR primers Ff/Rf (Fig. 5H). Thus, we can assume *ACTN2* is a direct target gene of RelA/NF-κB signaling in GC cells.

### NF-κB-triggered α-Actinin-2 positive auto-regulatory loop enhances *ACTN2* gene transcription

**Fig. 6:**
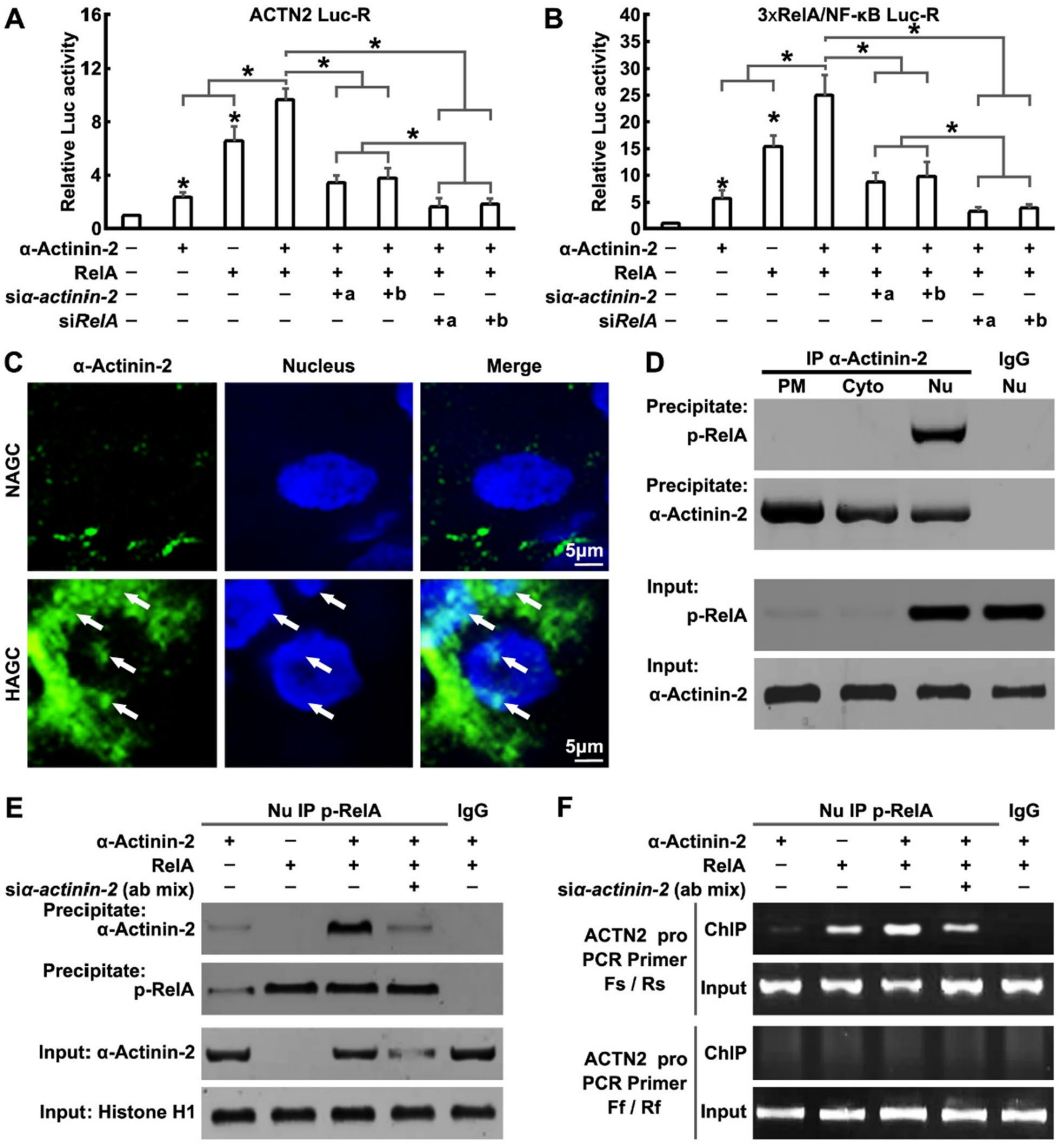
NF-κB-triggered α-Actinin-2 positive auto-regulatory loop enhances *ACTN2* gene transcription. **A** and **B** α-Actinin-2, RelA, α-Actinin-2 siRNAs and/or RelA siRNAs were cotransfacted with (**A**) *ACTN2* Luc-R or (**B**) 3xRelA/NF-κB Luc-R into SNU-16 cells for 12 h, then dual-luciferase reporter assays were performed. The statistical analysis information in A, B: The bars indicate the SD. The results are expressed as the mean ± SD of three independent experiments. *P < 0.05 using Ordinary one-way ANOVA. **C** NAGC and HAGC tissue sections obtained after infusion were immunostained for α-Actinin-2 (green) and nucleus (blue). **D** AGS cells were transfected with α-Actinin-2 for 24 h, then the PM, Cyto and Nu of cells were separated by the Subcellular Protein Fractionation Kit. IP assays were performed in PM, Cyto and Nu by using α-Actinin-2 antibodies. **E** and **F** α-Actinin-2, RelA and/or α-Actinin-2 siRNAs were cotransfacted into SUN-16 cells for 24 h, then cell nuclear extracts were subjected to ChIP analysis using the p-RelA antibody. **E** Western blotting analysis with the p-RelA antibody demonstrates the IP specificity and efficiency. **F** DNA isolated and purified from immunoprecipitated material was amplified with primers spanning the RelA binding site (Fs/Rs) or primers far away from the RelA binding site (Ff/Rf) of the *ACTN2* gene promoter

Interestingly, over-expression of full-length α-Actinin-2 only caused weak activation of the *ACTN2* promoter or RelA response reporter genes (Fig. 6A and B). However, co-expression of α-Actinin-2 with RelA enhanced while knockdown of α-Actinin-2 significantly decreased luciferase activity, compared to RelA alone, and knockdown of RelA could almost completely block α-Actinin-2 and RelA induced luciferase activity (Fig. 6A and B). On the other side, we found that α-Actinin2 co-overexpression with Rel-A has a promoting effect on the relative mRNA levels of the known RelA targets IL-6 and IL-8, but has an inhibitory effect on IL-1 and IL-2 (Additional file 1: Fig. S6C). Although α-Actinin2 and RelA together do not affect the expression of the ACTN2 gene only, these data also indicated that α-Actinin-2 specifically co-activated RelA and may potentially promote a positive auto-regulatory loop of its own transcription.

The function of α-Actinin-2 as a transcriptional co-activator must be associated with nuclear distribution. In this study, regional or punctate nuclear localization of α-Actinin-2 was observed in HAGC tissue cells and α-Actinin-2-overexpressing cells (Figs. 2C and 6C). By extracting nuclear protein from cells, we confirmed α-Actinin-2 localized and formed complexes with p-RelA in the nuclei (Fig. 6D).

To determine whether auto-regulation of α-Actinin-2 through the *ACTN2* gene is direct or indirect, a ChIP assay was used to study GC cells in the presence or absence of RelA overexpression, α-Actinin-2 overexpression, and/or α-Actinin-2-specific siRNAs. Chromatin was precipitated with a RelA antibody. α-Actinin-2 and RelA protein that had cross-linked to DNA was readily detected in precipitates (Fig. 6E). We found that the DNA fragments surrounding the RelA binding site could be amplified from RelA-overexpressing cells. Co-expression of α-Actinin-2 enhanced, but α-Actinin-2-specific siRNAs decreased this amplification (Fig. 6F), confirming the existence of a positive auto-regulatory loop for α-Actinin-2 in NF-κB-triggered *ACTN2* gene transcription.

### α-Actinin-2 promotes primary tumor growth and BMM in vivo

**Fig. 7:**
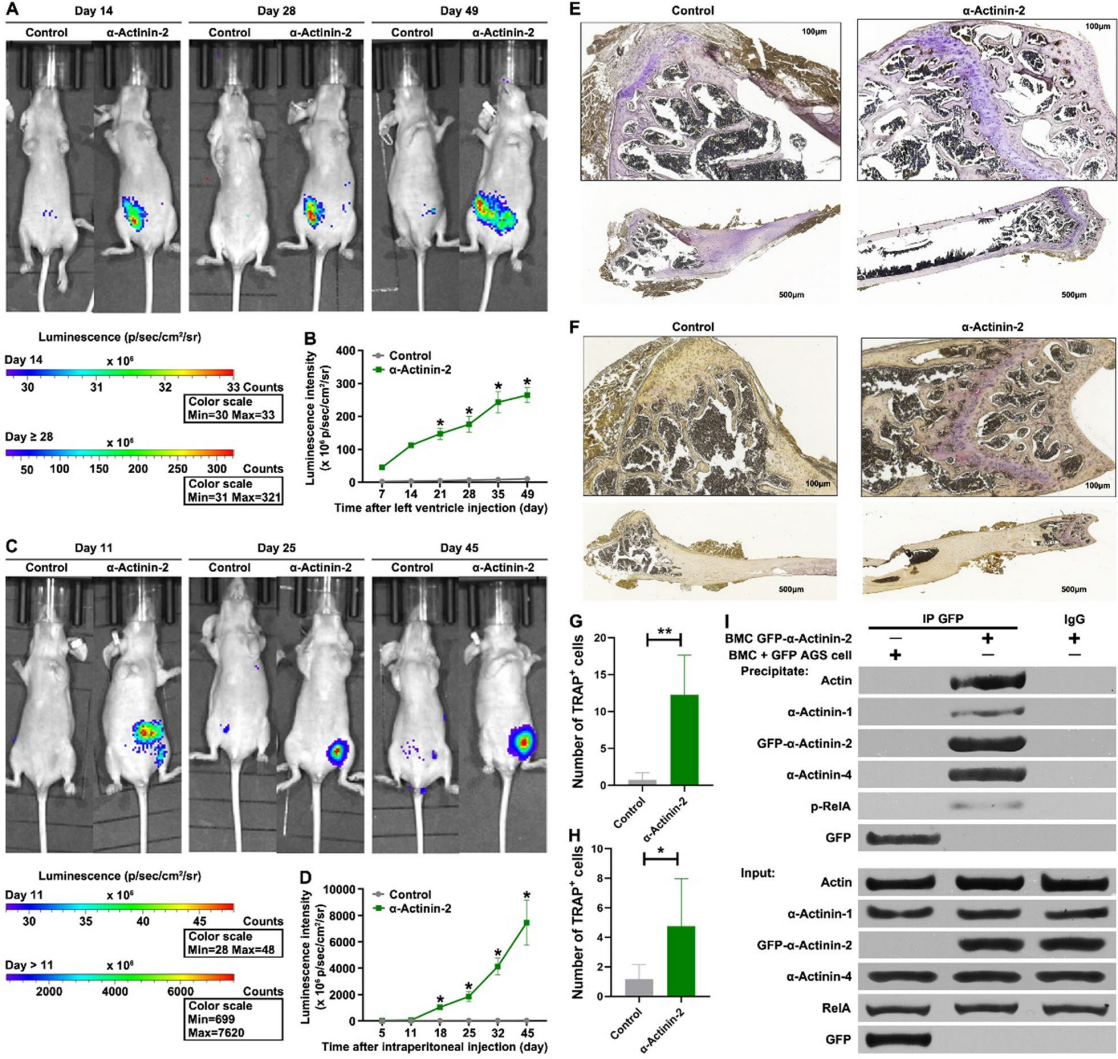
α-Actinin-2 promotes primary tumor growth and BMM in vivo. **A**, **B** 2 × 10^5^ luciferase-tagged and GFP or GFP-α-Actinin-2 transfected AGS cells were injected into the left cardiac ventricle of anesthetized female nude mice. Bioluminescence images show representative mice from each experimental group (A). Normalized BMM bioluminescence signals from mice (n = 7, B). The bars indicate the SD. The results are expressed as the mean ± SD of three independent experiments. *P < 0.05 using Ordinary one-way ANOVA. **C, D** 2 × 10^6^ luciferase-tagged and GFP or GFP-α-Actinin-2 transfected AGS cells were injected into the abdominal cavity of anesthetized female nude mice. Bioluminescence images show representative mice from each experimental group (**C**). Normalized BMM bioluminescence signals from mice (n = 11, **D**). **E–H** TRAP staining of femora. Representative images of cardiac injection and intraperitoneal injection mice. TRAP staining positive cells were indicated by black arrows. (**E**, **F**). Quantification of the number of TRAP staining positive cells in cardiac injection and intraperitoneal injection groups (**G**, **H**). The bars indicate the SD. The results are expressed as the mean ± SD of three independent experiments. *P < 0.05, **p < 0.01 using Student’s *t*-test. **I** Extracting mouse bone marrow cells from the site of bone metastasis, then the mouse bone marrow cells containing metastatic GFP-α-Actinin-2 overexpressed cancer cells or mouse bone marrow cells + GFP overexpressed AGS cells were lysed for co-Immunoprecipitation (IP) assay by using GFP antibodies

To recapitulate the in vitro findings and confirm the role of α-Actinin-2 in tumorigenesis and BMM in vivo, bioluminescent AGS cells (1 × 10^6^ cells) constitutively expressing α-Actinin-2 were injected into the left ventricle of athymic nude mice. Intracardiac injections in the left ventricle resulted in tumor cells initially being distributed throughout the entire arterial system and tested the ability of tumor cells to colonize all organs. Regular monitoring of mice with the IVIS imaging system was performed on a weekly basis. α-Actinin-2 significantly increased the primary tumor size as determined by bioluminescence imaging over the course of the experiment, compared to control (Fig. 7A). In view of the robust expansionary effect of α-Actinin-2 on GC cell migration in vitro, we reasoned that it may also promote metastasis in vivo. α-Actinin-2 induced spontaneous metastasis to bone 2 weeks after intracardiac injection whereas no metastasis was observed by the control cells (Fig. 7A and B). Interestingly, α-Actinin-2-overexpressing cells grew aggressively and metastatic lesions were observed in the pelvic and femoral bones but not in other organs (Fig. 7A and B), indicating the high efficiency and specificity of α-Actinin-2 in the induction of BMM of GC cells.

To confirm the BMM-promoting properties of α-Actinin-2, cells constitutively expressing α-Actinin-2 were injected intraperitoneally into athymic nude mice (Fig. 7C). Indeed, α-Actinin-2 induced metastasis to the bone only 11 days after intraperitoneal injection whereas no metastasis was observed for the control cells (Fig. 7A and B). Moreover, α-Actinin-2-overexpressing cells were also observed in the pelvic and femoral bones but not in other organs (Fig. 7C and D).

TRAP staining of osteoclasts was used to identify the α-Actinin-2-drived BMM. The TRAP positive cells in the α-Actinin-2 groups both of intracardiac injection mice and intraperitoneal injection mice increased compared to the control groups (Fig. 7E–H). The osteoclast distribution in the α-Actinin-2 intracardiac and the intraperitoneal injection groups were similar, but intracardiac injection recruited significantly more osteoclasts (Fig. 7G and H). Furthermore, by extracting mouse bone marrow cells from the site of bone metastasis, significant interactions between α-Actinin-2, α-Actinin-4 and Actin can still be observed in vivo (Fig. 7I). Meanwhile, the direct interaction between α-Actinin-2 and p-RelA was also observed in vivo (Fig. 7I). These results not only demonstrate that α-Actinin-2 was highly effective in promoting BMM but also showed that α-Actinin-2 could induce BMM independent to hematogenous metastasis.

## Discussion

Although the survival of patients with GC has significantly lengthened over the past decades, the five-year survival rate of metastatic GC remains only 5.5% at present [21]. In particular, the prognosis of patients with BMM is extremely poor with a median survival time of only 6.5 months [22]. Unfortunately, knowledge on the molecular mechanisms underlying BMM is scant. In this study, we report our findings on *ACTN2* gene, which showed that its expression was specifically increased in cancer tissues from HAGC with diffuse BMM, compared to NAGC and data from The Human Protein Atlas dataset. α-Actinin-2 played a vital role in bone metastases by facilitating cell filopodia formation through α-Actinin-2: α-Actinin-4 heterodimer generation. Here, we found that *ACTN2* was a direct target gene of the NF-κB signaling in GC cells and this NF-κB/α-Actinin-2 signaling pathway was boosted by a positive auto-regulatory loop of α-Actinin-2. Importantly, overexpression of α-Actinin-2 was sufficient to induce BMM in vivo. Therefore, these findings uncover a plausible mechanism for GC BMM and might provide the first therapeutic target for clinical intervention in HAGC.

BMM occurs when cancer cells invade surrounding tissue via an epithelial-to-mesenchymal transition, intravasate into the lymphatic or circulatory system and successfully extravasate into distant bone marrow. We found that α-Actinin-2-overexpression in GC cells enhanced cellular motility. This result is consistent with a very recent study on liver tumorigenesis in mice using the sleeping beauty transposon system [23] and confirms that α-Actinin-2 indeed plays a critical role in cancer cell metastasis. Cancer cells can pass through the extracellular matrix by releasing exosomes containing proteases such as matrix metalloproteinases at the tip of invadopodia [24]. We showed that α-Actinin-2 stimulated cancer cells gained mesenchymal phenotypes through fibroblast-like morphological changes, including filopodia formation. Filopodia play a central role in cell adhesion modulation through substrate tethering and environment sensing and many cell types use filopodia during the early spreading phase [10, 25, 26]. It has also been shown that exosomes surf on filopodia to enter cells [27] and filopodium-derived vesicles enhance the migration of recipient cells [13]. Sung et al*.* proposed a model in which exosomes secreted from cancer cells promote cancer cell chemotaxis in both an autocrine manner through secretion at the leading edge and a paracrine manner by leaving behind exosome trails [28]. In addition, we found that ectopic expression of α-Actinin-2 alone was sufficient to induce BMM of GC cells via both left ventricular intracardiac injection and intraperitoneal injection of nude mouse models. Moreover, α-Actinin-2 specifically induced BMM without causing metastasis to other distal tissues. Combined with our results and previous reports, we speculate that α-Actinin-2 is involved in the entire metastatic process of GC cells from in situ to bone through induction of filopodia formation.

Filopodia are thin, finger-like protrusions of the cytoskeleton composed of bundles of Actin filaments located at leading edges of cells and promote cell migration [10]. α-Actinins have a central effect in Actin cytoskeleton assembly in various cell types. In particular, α-Actinin-2 is the major Actin crosslinker in muscle Z-discs, focal adhesions, Actin stress fibers and neuronal spines. However, this Actin crosslinking ability of α-Actinin-2 has not been reported in cancer cells. It has been shown that α-Actinin-4 can alter the Actin cytoskeleton and enhance tumor cell metastasis by promoting the formation of invadopodia [29]. In other cases, it has been shown to exhibit tumor suppressor activity [30]. α-Actinin proteins typically form homo- or heterodimers to stabilize Actin networks at structures [14, 15]. Owing to this characteristic, the specific function of α-Actinin-4 may depend on its interacting partner. Foley et al*.* reported that α-Actinin-1 and α-Actinin-4 can form heterodimers and these heterodimers are more abundant than homodimers in several cancer cell lines [29]. We detected α-Actinin-1:α-Actinin-4 heterodimers in GC cells in the absence of α-Actinin-2 expression. However, it is clear that these heterodimers induced neither invadopodia nor filopodia in GC cells. Further, our results suggest α-Actinin-2 enhanced Actin filament cross-linking ability by replacing α-Actinin-1 to form α-Actinin-2:α-Actinin-4 complexes, and these new heterodimers promote F-Actin assembly into filopodia. Meanwhile, Liu X and Chu KM found α-Actinin-4 enhances migration and invasion of GC cells [31], these results are consistent with the phenomenon we have observed that α-Actinin-4 is required for α-Actinin-2-induced filopodia formation. However, we did not observe the upregulation of α-Actinin-4 in cancer tissue of patients with BMM, indicating that α-Actinin-2 but not α-Actinin-4 plays a dominant role in promoting the growth of filopodia and in inducing bone metastasis of cancer cells. Interestingly, a small amount of Tubulin also exist in the α-Actinin-2:α-Actinin-4:F-Actin complex (Fig. 4D), indicating a potential role of α-Actinin-2 in guiding microtubules into filopodia. Additionally, it is largely unknown how α-Actinin-2:α-Actinin-4 complexes mediate local clustering of Actin in the proximal parts of filopodia.

NF‐κB signaling has been reported to be a crucial contributor for cancer BMM via induction of local invasion and angiogenesis. Park et al*.* reported that NF‐κB plays a crucial role in the BMM of breast cancer by stimulating the expression of the gene encoding granulocyte macrophage-colony stimulating factor (GM-CSF) [32]. However, we detected a significant decrease in GM-CSF levels in the blood of patients with HAGC (data not shown). Using a series of cancer tissues from HAGC patients, we observed a concomitant increase of nuclear localized RelA with high α-Actinin-2 levels. We also confirmed that p-RelA and α-Actinin-2 formed heterotrimers in the nucleus and *ACTN2* is a direct target gene of this NF-κB complex in GC cells and in BMM mouse bone marrow cells, indicating a positive auto-regulatory loop involved in the transcription of the *ACTN2* gene. On one hand, NF-κB serves as a pivotal mediator of inflammatory responses [33]. On the other hand, chronic inflammation can promote tumor progression and BMM through signal regulation in and the microenvironment surrounding tumor cells. These data support a model in which NF-κB plays an important role in inflammation and oncogenic α-Actinin-2 signaling. Nevertheless, NF-κB is activated by many inflammatory stimuli and NF-κB binding sites have been identified in a wide variety of genes. Studies also found that inflammatory cytokines may function to inhibit tumor growth and development [34]. Similarly, Aksenova et al*.* demonstrated that α-Actinin-4 is a selective transcriptional co-activator of NF-κB to mediate the expression of matrix metalloproteinase genes MMP-3 and MMP-1 without affecting the expression of other NF-κB target genes, including BAX, TNC, FGF8, PTGS2, ICAM1 and FN1 in cancer cells [35]. Moreover, Zhao et al*.* identified α-Actinin-4 as a direct partner for NF-κB transcription factors in podocytes and showed that the nuclear function of α-Actinin-4 was independent of its Actin binding activity in the cytoplasm [36]. Consequently, selective disruption of RelA:α-Actinin-2 heterotrimers in the α-Actinin-2 positive feedback loop through inhibition of nuclear translocation of α-Actinin-2 may be a more practical strategy for preventing BMM.

In summary, our study delineated a novel role for α-Actinin-2 in promoting BMM in GC through filopodia formation. There are no reports identifying signaling pathways that regulate the BMM of GC to date. Thus, this discovery may provide insights into the causes of poor response of HAGC to treatment and provide evidence for new potential therapeutic targets. However, the direct molecular mechanism specifically underlying α-Actinin-2 induction of BMM remains unknown and requires further research in the future.

## Materials and methods

### Materials

Anti α-Actinin-1 (Cat No: PA5-17308), α-Actinin-2 (Cat No: 701914), α-Actinin-3 (Cat No: PA5-36233) and α-Actinin-4 (Cat No: MA5-32794) antibodies were from Invitrogen. Anti GFP (Cat No: ab290), Tubulin (Cat No: ab7291), Globular Actin (Cat No: ab205) and phospho-NF-κB (RelA)-Ser276 (Cat No: ab194726) antibodies were from Abcam. TRITC-conjugated-Phalloidine (Cat No: P1951) was form Sigma-Aldrich.

### Patients and clinical data

The database of the Sixth Affiliated Hospital of Sun-Yat Sen University (Guangzhou, China) was retrospectively inspected and all NAGC who were defined as GC without BMM based on routine staging workup and treated between January 2011 and December 2020 were included in this study. HAGC was identified as newly diagnosed GC with diffuse BMM which was defined as cases that had a BMM score ≥ 2, according to the extent of disease (EOD) criteria [37], on FDG‑PET/CTscan. All patients consented to the anonymous use of their medical information for research purposes on their first hospital visit. The study protocol was approved by the Sixth Affiliated Hospital of Sun-Yat Sen University clinical research ethics committee (Number 2021ZSLYEC-090). The authenticity of this article has been validated by uploading the key raw data onto the Research Data Deposit public platform (www.researchdata.org.cn). Patient demographics, serological, symptomatic data and outcomes were collected. Their histological slides and radiographical examinations were also retrieved and reviewed.

### Cell culture and plasmid transfections

The human GC cell lines of AGS and SUN-16 were purchased from ATCC and cultured in RPMI-1640 (Gibco) containing 10% fetal bovine serum (FBS; Gibco), penicillin (100 U/ml), and streptomycin (100 μg/ml) in a humidified incubator at 37 °C in 5% CO2. All cells were negative for mycoplasma. The cells were seeded in plates, and 24 h later were transfected with plasmid (detailed information in Additional file 1: Table S3) using Lipofectamine 2000, following the manufacturer’s protocols. The cells were harvested 24 h after transfection for real-time PCR analysis and 48 h after transfection for western blotting.

### siRNA transfections

si*α-actinin-1* a (siRNA ID: 147017), si*α-actinin-2* a (siRNA ID: 147020), si*α-actinin-4* a (siRNA ID: 147360) or si*RelA* a (siRNA ID: 109424) specific siRNAs were from Thermo Fisher Scientific and si*α-actinin-1* b (Cat No. sc-43095), si*α-actinin-2* b (Cat No. sc-43097), si*α-actinin-4* b (Cat No. sc-43101) or si*RelA* b (Cat No. sc-72100) specific siRNAs were from Santa Cruz Biotechnology. The negative control (NC) siRNA (no silencing small RNA fragment) was synthesized by GenChem Co. (Shanghai, China). siRNAs were transfected into cells using Lipofectamine RNAiMAX.

### RNA-seq and quantitative real-time PCR

Total RNA isolated from tumor tissues of HAGC and NAGC patients were subjected to paired-end RNA-Seq using Illumina HiSeq 2000 system according to the manufacturer’s instruction. RNA-seq data reads were mapped and assigned to the genes from Ensembl’s GRCh37/hg19 annotation using STAR and the feature Counts program from the Rsubread package (v1.20.6) in R. The fragment per kilobase of transcript per million (FPKM) was calculated using the fpkm function from the edgeR package (v 3.12.1). The log2 fold change was calculated with DESeq2 and differentially expressed genes of tumor tissue were identified with a log2 fold-change > 0.8–1 and *P* value < 0.05 compared to tumor-adjacent tissue. Gene Set Enrichment Analysis (GSEA, Broad Institute, http://www.broadinstitute.org/gsea/index.jsp) was performed using gene sets in the MSigDB database or published gene sets as indicated. Gene Ontology (GO) was conducted using DAVID. Cells were harvested in TRIzol reagent (Invitrogen), and total RNA was extracted. qRT-PCR was performed to determine the transcription level of a gene using SYBR Premix Ex Taq kit (TaKaRa), and its corresponding primer pair (Additional file 1: Table S3).

### Transwell migration assay

GC cells were plated at 1 × 10^5^ cells/well in 8.0-mm pore-sized 24-well Transwell inserts (Corning) with serum-free RPMI-1640, and the lower chamber was filled with 0.6 ml/well of RPMI-1640 supplemented with 2% FBS. The cells were incubated at 37 °C for 24 h. Thelower side of the filter was fixed in 4% paraformaldehyde (Electron Microscopy Science) and stained with 0.5% Crystal violet (Sigma-Aldrich) for 20 min, followed by examination under a microscope whereby 5 randomly non-overlapping high-power fields (HPFs, × 200) were selected to count the stained migrated cells. All the experiments in each group were performed in triplicate.

### Scratch wound healing assay

GC cells were seeded in 6-well plates and allowed to grow to ~ 90% confluence. The plates were scratched with a 200 μl pipette tip across the center of the well to create a straight line. The cells were washed twice with phosphate-buffered solution (PBS) to remove any detached cells, and fresh RPMI-1640 supplemented with 2% FBS was added to each well. After 24 h, images were captured under a microscope to assess the rate of gap closure.

### Western blotting

Proteins were separated by 8–10% SDS-PAGE, transferred to a PVDF membrane and incubated with primary antibodies. The signal was detected by Pierce™ ECL Western Blotting Substrate detection system.

### Subcellular fractionation

The Subcellular Protein Fractionation Kit (Thermo Fisher Scientific, #78840) was used following the manufacturer’s instructions. Briefly, the lysate of cultured cells is centrifuged to obtain the cytosolic fraction and the pellet containing membrane, organelles and nuclear extract including soluble and chromatin-bound nuclear extract.

### Dual-luciferase reporter assays and related constructs

The *ACTN2* luciferase porter (*ACTN2* Luc-R), containing nucleotides from − 1918/− 1427/− 907/− 412/+ 331/+ 616/+ 793 to + 2455 of the Human *ACTN2* gene (Gene ID: 88) relative to the transcription start site. The *ACTN2* Luc-R was cloned into the pGL3-basic vector. NF-κB transcription activity reporter plasmid was constructed by inserting a 3 × NF-κB binding sequence (GGGAATTTCCGGGAATTTCC) into the pGL3-basic vector. The mutations of the RelA binding site in *ACTN2* Luc-R were generated by QuikChange II Site-Directed Mutagenesis Kit. For the dual-luciferase reporter assays, the cells were transfected with 1 μg of a luciferase reporter plasmid and 200 ng of the pRL-CMV Renilla luciferase reporter plasmid. Firefly luciferase activity was normalized to Renilla luciferase activity according to the protocol of the Dual-Luciferase Reporter Assay System and our previous study [38].

### Multiple immunofluorescence staining

GFP-tagged α-Actinin-2 were overexpressed in GC cells. The cells were fixed with 4% paraformaldehyde, permeabilized with 0.3% triton, followed by incubation with anti-α-Actinin-1 (1:250), anti-α-Actinin-4 (1:250) and secondary antibodies conjugated with Alexa Fluor 405 or 647. F-Actin was stained with TRITC-conjugated-Phalloidine (1:800). Paraffin-embedded tumor tissue sections of HAGC and NAGC patients were incubated with the following: anti-Phospho-NF-κB (RelA)-Ser276 (1:100), anti-α-Actinin-2 (1:250), and secondary antibodies conjugated with Alexa Fluor 488 and 647. Nuclei were counterstained with DAPI (1 μg/mL). Images were acquired on a Zeiss LSM780 confocal microscope. Fluorescence colocalization analysis was performed using the ImageJ Plugins software according to previous study [39].

### Filopodia quant analysis

For analysis of filopodia, optical sections at 4 μm near the bottom area of the cells were captured. FiloQuant plugin for the ImageJ software (1.52i) was utilized according to previous studies [40]. Single image FiloQuant was used to detect and measure the length and number of filopodia.

### G-Actin and F-Actin ratio assays

G-Actin and F-Actin ratio assays were raised following the methodology of a previous study [41] and implemented through the G-Actin/F-Actin In Vivo Assay Kit (Cat. # BK037, Cytoskeleton, Inc.). Briefly, the cells were washed three times in 37 °C PBS (pH 7.4) before lysis with Actin stabilization buffer (0.1 M PIPES pH 6.9, 30% glycerol (vol/vol), 5% DMSO (vol/vol),1 mM MgSO_4_, 1 mM EGTA, 1% Triton X-100 (vol/vol), 1 mM ATP, complete protease inhibitor and phosphatase inhibitor. The lysis was kept at 37 °C for 10 min. Cells were dislodged by scraping and the entire extract centrifuged at 37 °C for 75 min at 100,000 *g*. The supernatant containing G-Actin was recovered and the pellet containing F-Actin collected separately and resuspended with RIPA buffer. Samples of the supernatant and pellets were run in an SDS-PAGE system and Actin was quantitated by Western blotting analysis.

### Co-immunoprecipitation

Co-Immunoprecipitation assays were implemented through the Pierce Direct IP Kit. This IP Kit uses an activated resin to covalently immobilize IP antibodies on agarose beads without the aid of Protein A/G, enabling immunoprecipitation without antibody interference. Briefly, cell extracts were prepared by solubilizing 10^7^ cells in 1 ml of cell lysis buffer at 4 °C. After brief sonication, the lysates were cleared by centrifugation at 15,000 *g* for 10 min at 4 °C. The cell extracts or proteins were immunoprecipitated with 2 μg of immobilized IP antibodies and incubated for 6 h at 4 °C by continuous inversion. Immunocomplexes were pelleted, washed 4 times, boiled in Laemmli buffer and analyzed by Western blotting.

### In vitro G-Actin and F-Actin binding studies

G-Actin and F-Actin were reconstituted in water to achieve a stock concentration of 0.4 mg/ml in a buffer that consisted of 5 mM Tris–HCl (pH 8.0), 0.2 mM CaCl_2_, 0.2 mM ATP, 2 mM MgCl_2_, and 5% (wt/vol) sucrose as directed by the supplier. This solution was stored at − 80 °C in 50 μl (20 μg) aliquots until use. Immediately before use, each aliquot was thawed by placing it in a 37 °C water bath for 5 min and then at room temperature. Human α-Actinin-1/2/4 proteins were expressed in a Mammalian Expression System and prepared by Detai Biologics (Nanjing, China). For all studies, 10 μg of G-Actin or F-Actin was used to incubate with 10 μg α-Actinin-1/10 μg α-Actinin-4 or 10 μg α-Actinin-2/10 μg α-Actinin-4 for 15 min in 100 μl reaction buffer [5 mM Tris–HCl (pH 8.0), 0.2 mM ATP, and 2 mM MgCl_2_] at room temperature, then were immunoprecipitated with 5 μg of immobilize IP antibodies and performed using the Pierce Direct IP Kit. Immunocomplexes were pelleted, washed 4 times, boiled in Laemmli buffer and analyzed by Western blotting.

### Chromatin immunoprecipitation assays (ChIP)

ChIP assays were performed using the SimpleChIP Enzymatic Chromatin IP Kit according to the manufacturer’s instructions and our previous study [38]. The immunoprecipitation was finished by using the Pierce Direct IP Kit. Purified DNA was subjected to PCR amplification using primers spanning the *ACTN2* gene promoter RelA site (forward Fs: 5′-TTG TGT CCA TTT ACC CCA GCC-3′, reverse Rs: 5′-CGT CCC TGT GTG CTT ACC TCA-3′); or primers far away from the *ACTN2* gene promoter RelA site as negative control (forward Ff: 5′-CCG CTT GCC CTT CAT TGT CAG-3′, reverse Rf: 5′-TTT CTC TCC TCC TCA CCC TCA C-3′).

### Animal models for BMM assay

All animal procedures and care were approved by the Institutional Animal Care and Usage Committee of Sun Yat-Sen University (No. L102012018050D). The animal model was established as previously described [42]. For BMM studies, 2 × 10^5^ luciferase-tagged AGS cells were injected into the left cardiac ventricle of anesthetized female nude mice. Similarly, 2 × 10^6^ luciferase-tagged AGS cells were intraperitoneally injected. Development of bone metastases was monitored by measuring photon flux of BLI signals in the hindlimbs of mice after intraperitoneal injection of 75 mg/kg D-Luciferin (PerkinElmer, #122799). Bioluminescence images were acquired with the IVIS Imaging System (PerkinElmer) at 2–5 min after injection. BLI signal data were acquired after background subtraction. Data were normalized to the signal obtained immediately after xenografting (day 0).

### Bone histological analysis

Femora were fixed with 4% formalin, decalcified in 10% EDTA for 2 weeks and subsequently embedded in paraffin. Hematoxylin & eosin (H&E) staining of the Sects. (5 μm thick) were performed according to standard procedures. Tartrate-resistant acid phosphatase (TRAP) staining was performed using the Leukocyte Acid Phosphatase Assay (Sigma-Aldrich) following the manufacturer’s protocol. The number of TRAP-positive cells per millimeter of trabecular bone perimeter was quantified in the secondary spongiosa.

### Statistical analysis

Categorical data were presented as the numbers (percentage), and differences between HAGC and NAGC patients were analyzed using the Chi-square tests. Time-to-event endpoints were compared using the non-stratified log-rank test. Kaplan–Meier estimates and Cox regression analyses of overall survival were also performed. The log-rank test was used to compare the distribution between HAGC and NAGC. For the subgroup analysis of overall survival, the HR and 95% CI within each subgroup were summarized. All analyses were performed using the IBM SPSS (version 26.0) and GraphPad Prism (8.0 version) software. Statistical significance was considered for data with two-sided *P* < 0.05 for all tests.

## Supplementary Information

**Additional file 1: Figure S1.** The promotion of gastric cancer cell motility by ACTN2 was independent of other genes. Cell migration and invasion were detected when AGS cells were co-transfected with *ACTN2* and *ADIPOQ* (A), and SNU-16 cells were co-transfected with *ACTN2* and *SYT12*/*SPINK6* (B, C). The bars indicate the SD. The results are expressed as the mean ± SD of five independent experiments. *p < 0.05, **p < 0.01 using Ordinary one-way ANOVA. ns, no significance. **Figure S2.** (A) α-Actinin-2 promotes GC cell migration by facilitating the increase and growth of filopodia. SNU-16 cells were transfected with GFP or GFP-α-Actinin-2 for 24 h, then cells were stained for DAPI (Nucleus, blue), GFP/GFP-α-Actinin-2 (green) and F-Actin (red). (B-E) Overexpression of α-actinin-2 had no influence of cell viability and apoptosis. (B) Cell viability were detected when AGS/ SNU-16 cells were transfected with ACTN2 from day 1 to 7. (C) Clone formation assay in AGS and SNU-16 cells with stable ACTN2 overexpression. (D) Cell cycle data were measured by flow cytometry in AGS and SNU-16 cells with stable ACTN2 overexpression. (E) Cell apoptosis were detected by Annexin V/PI assay after transfection with ACTN2. The bars indicate the SD. The results are expressed as the mean ± SD of five independent experiments. **Figure S3.** **(A)** The identify of N-terminal and C-terminal amino acid sequence between α-Actinin 1, 2, 3 and 4.** (B)** GFP-α-Actinin 1, 2, 3 or 4 was overexpressed in AGS cells for 24 h, then cell lysates were harvested and subjected to Western blotting using indicated antibodies. **(C)** AGS cells were transfected with α-Actinin-2 for 24 h, then the membrane extract (PM), cytoplasmic extract (Cyto) and nuclear extract (Nu) of cells were separated by the Subcellular Protein Fractionation Kit. Glut1, the protein marker of PM; Tubulin, the protein marker of Cyto; Histone H1, the protein marker of Nu. **(D)** GFP-α-Actinin-2 was co-transfected with or without α-Actinin-2 siRNAs in AGS cells for 24 h, then cells were treated as in (Figure 3B) to separate the supernatant and pellet. The pellet was used for co-Immunoprecipitation (IP) assay by using α-Actinin-2 antibodies. **Figure S4. (A) **α-Actinin-2 was co-transfected with or without α-Actinin-1 siRNAs in AGS cells for 24 h, then cells were lysed for co-Immunoprecipitation (IP) assay by using α-Actinin-2 antibodies. **(B and C) In vitro** G-Actin/F-Actin and α-Actinin-1:α-Actinin-4 complex or α-Actinin-2:α-Actinin-4 complex binding studies were performed and analyzed by Western blotting using the indicated antibodies, **(B)** and **(C)** show inputs of the experiment. **Figure S5.** NF-κB pathway was activated in HAGC. **(A, B)** GO analysis with differentially expressed genes in NAGC vs normal as well as HAGC vs normal. **(C)** Pathway specifically enriched in HAGC by GO analysis. **(D) **Heatmap representing differential expressed genes (fold change > 2) which were transcriptional targets of RelA in HAGC and NAGC. **Figure S6. (A) **AGS cells were transfected with GFP-RelA for 2 days following transfect with negative control (NC) siRNAs or α-Actinin-2 siRNAs for 24 h, then cells were stained for quantification of the number of filopodia per cell or the average length of filopodia. GFP/GFP-α-Actinin-2 (green), F-Actin (red) and Nucleus (DAPI, blue). Scale bar, 10 μm. (**B**) GFP-RelA was co-transfected with or without α-Actinin-2 siRNAs in AGS cells for 24 h, then the ratio of F-Actin/G-Actin was determined by G-Actin and F-Actin ratio assays. P: pellet; S: supernatant. (C) AGS cells were transfected with RelA, α-Actinin-2 or RelA plus α-Actinin-2 for 2 days following transfect with negative control (NC) siRNAs or α-Actinin-2 siRNAs for 24 h, then the total RNA of cell were extracted for qRT-PCR analysis. The bars indicate the SD. The results are expressed as the mean ± SD of three independent experiments. *P < 0.05 using Ordinary one-way ANOVA for (A and B). *P < 0.05 using Student’s *t*-test for (C). **Table S1.** Clinical characteristics of patients with HAGC and NAGC. **Table S2.** Up-regulated genes in HAGC and/or NAGC. **Table S3.** Plasmids and qRT-PCR primers. **Table S4.** Clinical characteristics of patients detected by multiple immunofluorescence staining of α-Actinin-2 and p-RelA.

## Data Availability

The data that support the findings of this study are available from the corresponding author upon reasonable request.
