# Supplementary material for: Induction of filopodia formation by α-Actinin-2 via RelA with a feedforward activation loop promoting overt bone marrow metastasis of gastric cancer
Source: J Transl Med. 2023 Jun 19;21:399. doi: 10.1186/s12967-023-04156-w (PMC10280853; doi:10.1186/s12967-023-04156-w)
Supplement: Supplementary file 1 — Additional file 1: Figure S1. The promotion of gastric cancer cell motility by ACTN2 was independent of other genes. Cell migration and invasion were detected when AGS cells were co-transfected with ACTN2 and ADIPOQ (A), and SNU-16 cells were co-transfected with ACTN2 and SYT12/SPINK6 (B, C). The bars indicate the SD. The results are expressed as the mean ± SD of five independent experiments. *p < 0.05, **p < 0.01 using Ordinary one-way ANOVA. ns, no significance. Figure S2. (A) α-Actinin-2 promotes GC cell migration by facilitating the increase and growth of filopodia. SNU-16 cells were transfected with GFP or GFP-α-Actinin-2 for 24 h, then cells were stained for DAPI (Nucleus, blue), GFP/GFP-α-Actinin-2 (green) and F-Actin (red). (B-E) Overexpression of α-actinin-2 had no influence of cell viability and apoptosis. (B) Cell viability were detected when AGS/ SNU-16 cells were transfected with ACTN2 from day 1 to 7. (C) Clone formation assay in AGS and SNU-16 cells with stable ACTN2 overexpression. (D) Cell cycle data were measured by flow cytometry in AGS and SNU-16 cells with stable ACTN2 overexpression. (E) Cell apoptosis were detected by Annexin V/PI assay after transfection with ACTN2. The bars indicate the SD. The results are expressed as the mean ± SD of five independent experiments. Figure S3. (A) The identify of N-terminal and C-terminal amino acid sequence between α-Actinin 1, 2, 3 and 4. (B) GFP-α-Actinin 1, 2, 3 or 4 was overexpressed in AGS cells for 24 h, then cell lysates were harvested and subjected to Western blotting using indicated antibodies. (C) AGS cells were transfected with α-Actinin-2 for 24 h, then the membrane extract (PM), cytoplasmic extract (Cyto) and nuclear extract (Nu) of cells were separated by the Subcellular Protein Fractionation Kit. Glut1, the protein marker of PM; Tubulin, the protein marker of Cyto; Histone H1, the protein marker of Nu. (D) GFP-α-Actinin-2 was co-transfected with or without α-Actinin-2 siRNAs in AGS cells for 2 [file 12967_2023_4156_MOESM1_ESM.docx]

**Supplementary Materials**

| **Part one: Materials and methods** |
| --- |
| Reagents |
| Cell proliferation analysis |
| Flow cytometry to detect the cell cycle and apoptosis |
| Mouse Bone Marrow Cell Isolation |
|  |
| **Part two:**  **Supplementary Figures and Figure Legends** |
| Fig. S1 |
| Fig. S2 |
| Fig. S3 |
| Fig. S4 |
| Fig. S5 |
| Fig. S6 |
|  |
| **Part three: Tables** |
| Table S1 Clinical characteristics of patients with HAGC and NAGC |
| Table S2 Up-regulated genes in HAGC and/or NAGC |
| Table S3 Plasmids and qRT-PCR primers |
| Table S4 Clinical characteristics of patients detected by multiple immunofluorescence staining of α-Actinin-2 and p-RelA |

**Part one:** **Materials and methods**

**Reagents**

DAPI (Cat No. D9542) was obtained from Sigma-Aldrich. Invitrogen Lipofectamine 2000 (Cat No. 11668019), Lipofectamine RNAiMAX (Cat No. 13778150), Pierce Direct IP Kit (Cat No. 26148), Pierce ECL Western Blotting Substrate (Cat No. 32106) and Subcellular Protein Fractionation Kit for Cultured Cells (Cat No. 78840) were obtained from Thermo Fisher Scientific. G-actin/F-actin In Vivo Assay Kit (Cat No. BK037), Purified rabbit skeletal muscle F-Actin (Cat No. AKF99) and G-Actin (Cat No. AKL95) were purchased from Cytoskeleton, Inc. QuikChange II Site-Directed Mutagenesis Kit (Cat No. 200523) was obtained from Agilent. Dual-Luciferase Reporter Assay System (Cat No. E1980) was obtained from Promega (Beijing) Biotech Co., Ltd. Tetramethyl rhodamine isothiocyanate (TRITC)-conjugated Phalloidin (Cat No. CA1610), Anti-Phospho-NF-κB (RelA)-Ser276 Polyclonal antibody (Cat No. K006209P), Anti-α-Actinin-1 antibody (Cat No. K002308P), Anti-α-Actinin-2 antibody (Cat No. K009726P), Anti-α-Actinin-3 antibody (Cat No. K002675P), Anti-α-Actinin-4 antibody (Cat No. K107010P) were obtained from Solarbio Life Science. Anti-α-Actinin-2 antibody (Cat No. GTX632361) was obtained from GeneTex, Inc. SimpleChIP Enzymatic Chromatin IP Kit (Cat No. 9002), Anti-Actin antibody (Cat No. 4968), Anti-GAPDH antibody (Cat No. 2118), Anti-Histone H1 antibody (Cat No. 41328) and Anti-β-Tubulin antibody (Cat No. 2146) were obtained from Cell Signaling Technology, Inc. Goat Anti-Rabbit IgG H&L (Alexa Fluor 405, Cat No. ab175652), Donkey Anti-Rabbit IgG H&L (Alexa Fluor 488, Cat No. ab150073) and Donkey Anti-Rabbit IgG H&L (Alexa Fluor 647, Cat No.ab150075) were obtained from Abcam. Anti-Glut1 antibody (Cat No. NB110-39113) was obtained from Bio-Techne China Co. Ltd.

**Cell proliferation analysis**

For colony formation assay, a total of 300 melanoam cells were seeded in a 6-well plate and cultured in complete DMEM medium for 10 days. Colonies were fixed with 4% polymethanol and dyed with 0.1% crystal violet (1 mg/mL), and the number of colonies with over 50 cells was counted. For CCK-8 assay , a total of 300 melanoam cells were seeded in triplicate in each well of a 96-well plate, and the cell numbers were counted every day by CCK-8 (No. CK04-500, DOJINDO, Kumamoto, Japan) for 7 days.

**Flow cytometry to detect the cell cycle and apoptosis**

AGS and SNU-16 cells with stable ACTN2 overexpression were plated and grown in culture plates, and then collected and processed for analysis using flow cytometry to detect cell cycle and apoptosis. For cell cycle analysis, the cells were fixed and stained with propidium iodide (PI), while for apoptosis analysis, they were stained with Annexin V-PE and 7-AAD. The experiments were repeated three times.

**Mouse bone marrow cell isolation**

Mouse bone marrow cell isolation was raised following the methodology of previous studies [1, 2]. Sacrifice a mouse by cervical dislocation and thoroughly soak with 70% ethanol. Prepare a petri dish with ice-cold DPBS (Sterile, ice-cold Dulbecco’s phosphate buffered saline). Using sterile scissors and forceps, make an incision in the skin where the leg meets the hip and cut all the way around the leg. Peel skin down to the foot and remove. Carefully remove the leg by cutting at the hip joint, making sure to keep the femur intact. Surrounding muscle can be removed as needed to better visualize the joint. Remove foot and place leg in the petri dish containing DPBS. Repeat with the second leg. Prepare a fresh petri dish containing ice-cold DPBS. Remove muscle tissue (and fibula from tibia) from bones and carefully separate femur from tibia, keeping both bones intact. Dip cleaned bones in 70% ethanol and place in a petri dish. Fill two 10 ml syringes with ice-cold DPBS and attach 25G needles. With a fresh pair of sterile forceps, remove one bone from the petri dish and use a fresh pair of sterile scissors to remove the epiphyses. Holding the bone vertically over an open 50 ml tube on ice, insert the needle into the bone cavity and flush the marrow from the bone with the DPBS into the 50 ml tube. Invert the bone and repeat, running the needle up and down through the cavity to dislodge any remaining marrow. Repeat this process with the remaining bones. Pipet the marrow suspension up and down with a 10 ml pipette to break up the marrow and filter through a 70 μm cell strainer to separate out any remaining clumps. Centrifuge the cells for 10 min at 500 × g, 4 °C. Remove supernatant and resuspend cells in 10 ml 1× red blood cell lysis buffer (eBioscience™, Cat No.: 00-4333-57). Incubate for 2 min at room temperature. Add 20 ml ice-cold DPBS and centrifuge for 10 min at 500 × g, 4 °C. The resulting pellet should be white. Resuspend the cells in 20 ml warm complete media and determine cell concentration with hemocytometer or automated cell counter. Typical yield can range from 3.5 × 10^7^ to 5 × 10^7^ cells/mouse.

References:

[1] Liu X, et al., Immune Cell Isolation from Mouse Femur Bone Marrow. *Bio Protoc.* 2015;5(20):e1631. PMID: 27441207

[2] Mendoza R, et al., Mouse Bone Marrow Cell Isolation and Macrophage Differentiation. *Methods Mol Biol.* 2022; 2455:85-91. PMID: 35212988

**Part two: Supplementary Figures** **and Figure Legends**


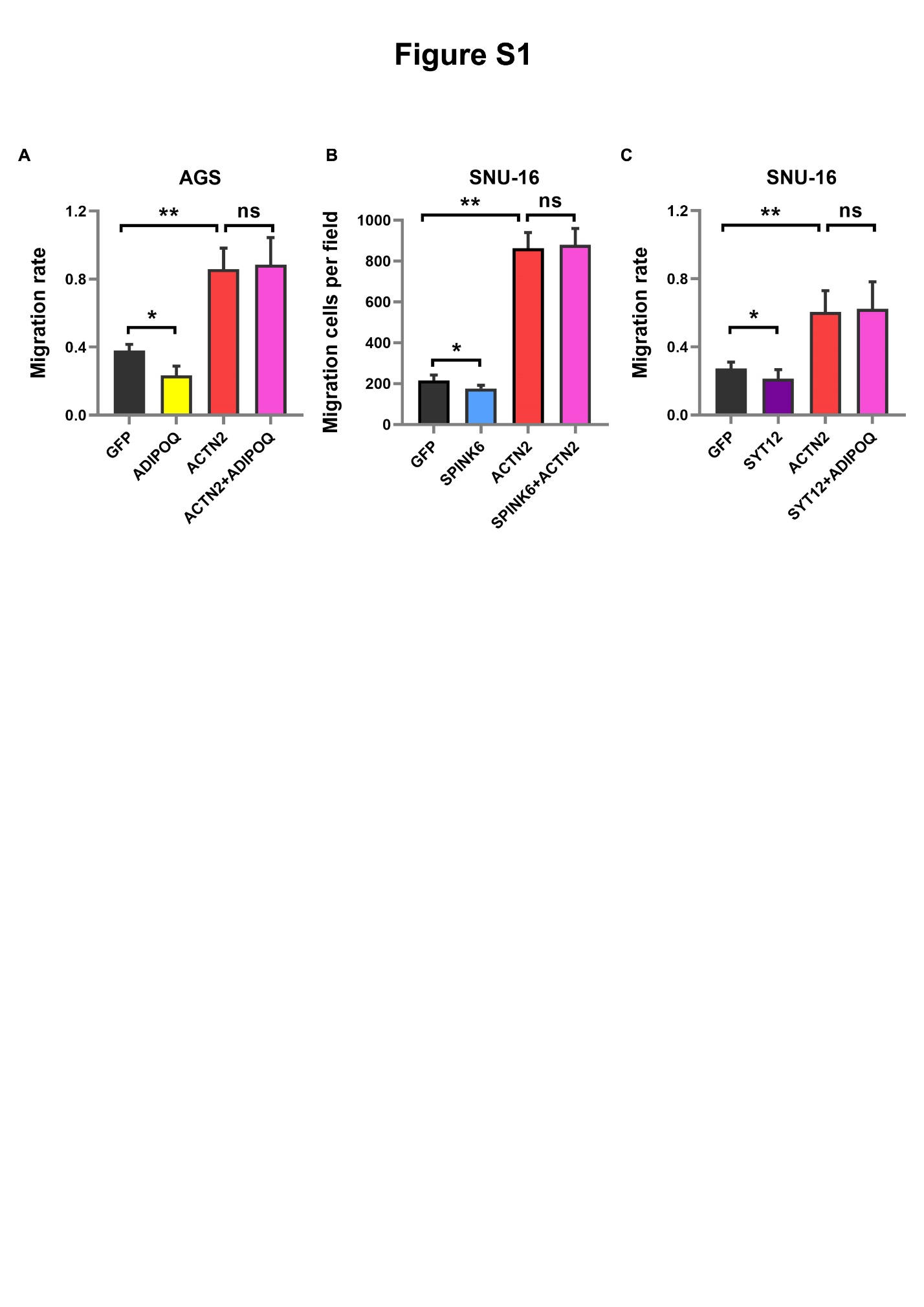


**Fig. S1 The promotion of gastric cancer cell motility by ACTN2 was independent of other genes.** Cell migration and invasion were detected when AGS cells were co-transfected with *ACTN2* and *ADIPOQ* (A), and SNU-16 cells were co-transfected with *ACTN2* and *SYT12*/*SPINK6* (B, C).  The bars indicate the SD. The results are expressed as the mean ± SD of five independent experiments. *p < 0.05, **p < 0.01 using Ordinary one-way ANOVA. ns, no significance.


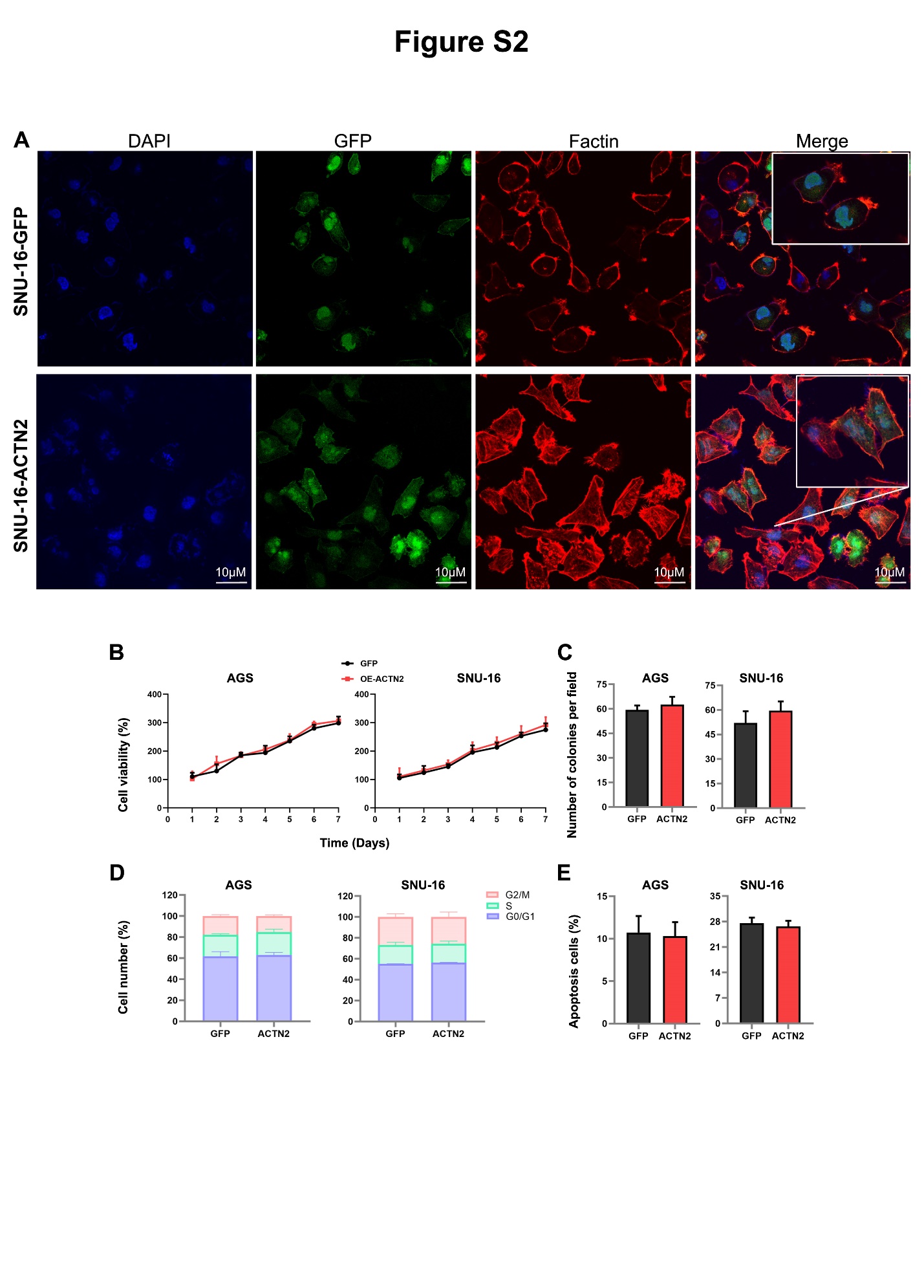


**Fig. S2 (A) α-Actinin-2 promotes GC cell migration by facilitating the increase and growth of filopodia.** SNU-16 cells were transfected with GFP or GFP-α-Actinin-2 for 24 h, then cells were stained for DAPI (Nucleus, blue), GFP/GFP-α-Actinin-2 (green) and F-Actin (red). **(B-E) Overexpression of α-actinin-2 had no influence of cell viability and apoptosis.** (B) Cell viability were detected when AGS/ SNU-16 cells were transfected with ACTN2 from day 1 to 7. (C) Clone formation assay in AGS and SNU-16 cells with stable ACTN2 overexpression. (D) Cell cycle data were measured by flow cytometry in AGS and SNU-16 cells with stable ACTN2 overexpression. (E) Cell apoptosis were detected by Annexin V/PI assay after transfection with ACTN2. The bars indicate the SD. The results are expressed as the mean ± SD of five independent experiments.

**
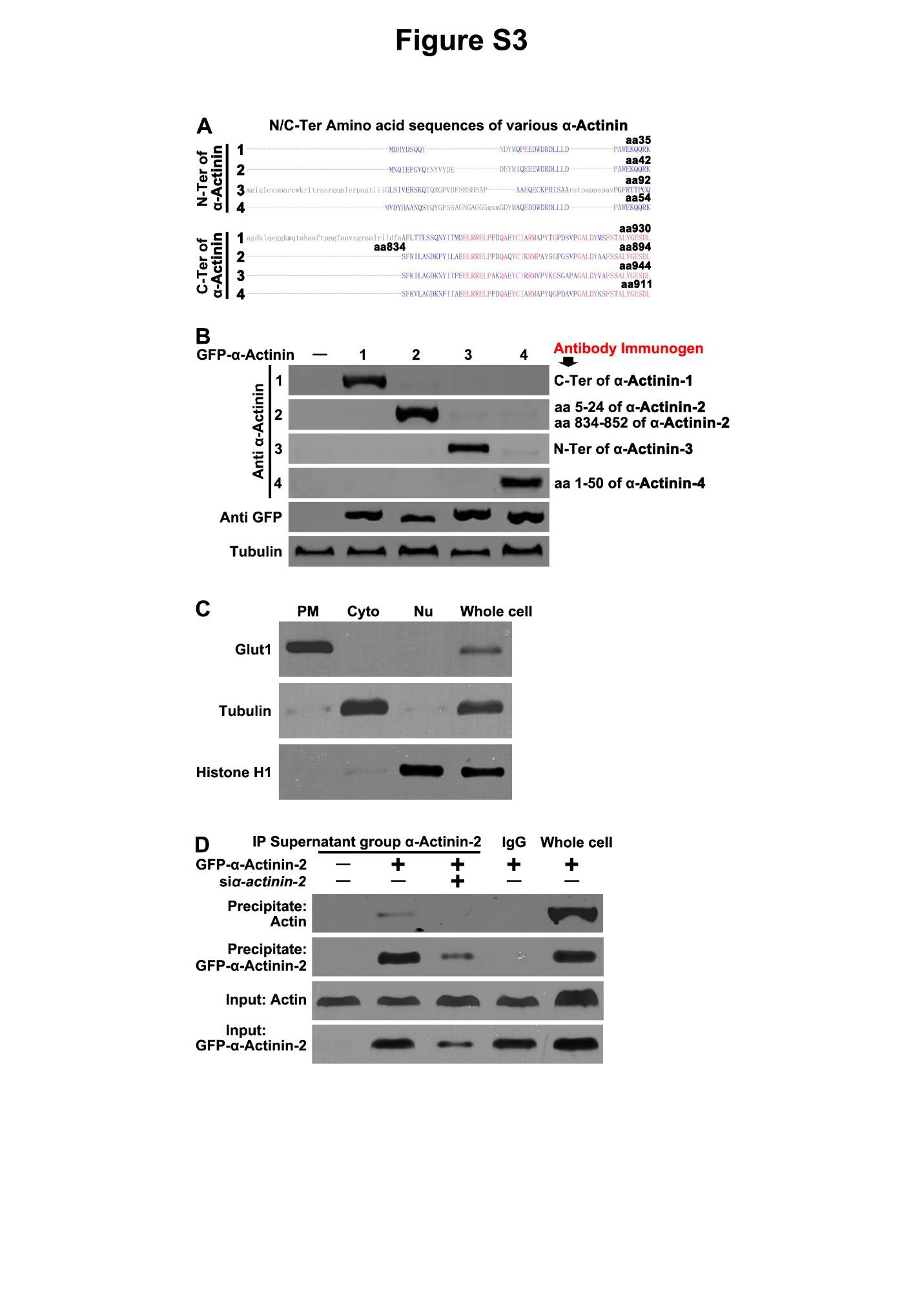
**

**Fig. S3** **(A)** The identify of N-terminal and C-terminal amino acid sequence between α-Actinin 1, 2, 3 and 4. **(B)** GFP-α-Actinin 1, 2, 3 or 4 was overexpressed in AGS cells for 24 h, then cell lysates were harvested and subjected to Western blotting using indicated antibodies. **(C)** AGS cells were transfected with α-Actinin-2 for 24 h, then the membrane extract (PM), cytoplasmic extract (Cyto) and nuclear extract (Nu) of cells were separated by the Subcellular Protein Fractionation Kit. Glut1, the protein marker of PM; Tubulin, the protein marker of Cyto; Histone H1, the protein marker of Nu. **(D)** GFP-α-Actinin-2 was co-transfected with or without α-Actinin-2 siRNAs in AGS cells for 24 h, then cells were treated as in (Figure 3B) to separate the supernatant and pellet. The pellet was used for co-Immunoprecipitation (IP) assay by using α-Actinin-2 antibodies.


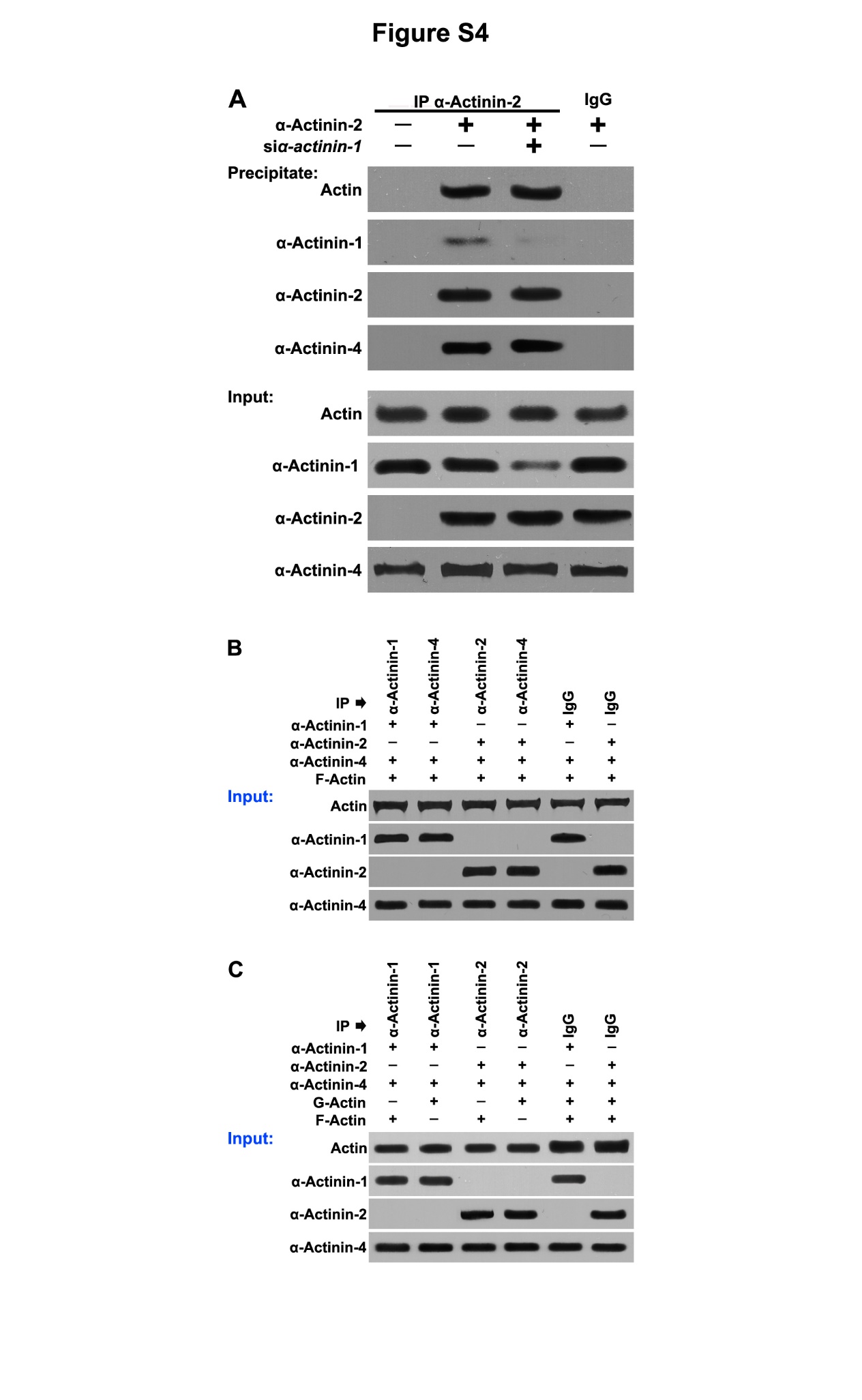


**Fig. S4 (A)** α-Actinin-2 was co-transfected with or without α-Actinin-1 siRNAs in AGS cells for 24 h, then cells were lysed for co-Immunoprecipitation (IP) assay by using α-Actinin-2 antibodies. **(B and C)** *In vitro* G-Actin/F-Actin and α-Actinin-1:α-Actinin-4 complex or α-Actinin-2:α-Actinin-4 complex binding studies were performed and analyzed by Western blotting using the indicated antibodies, **(B)** and **(C)** show inputs of the experiment.


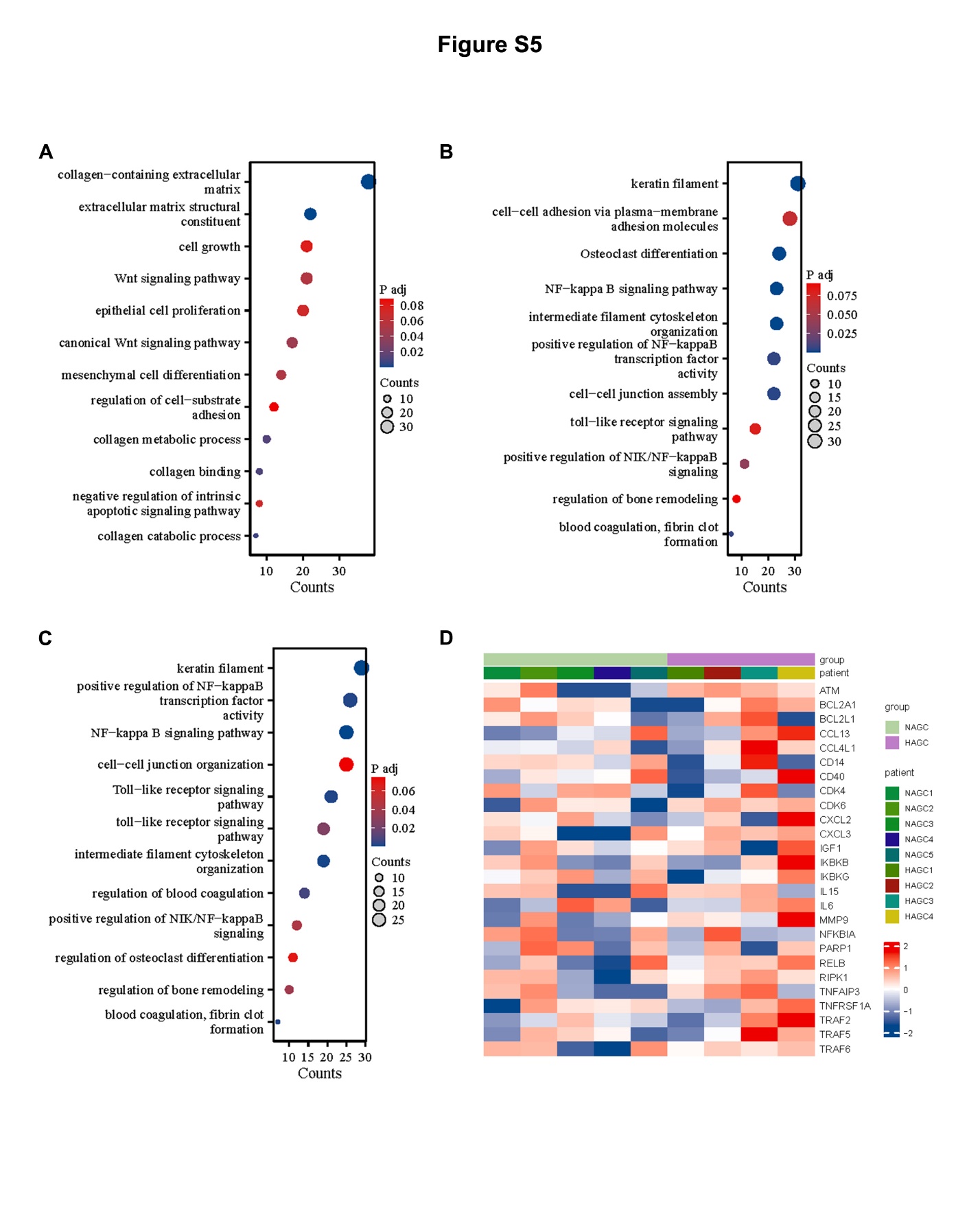


**Fig. S5** **NF**-κB **pathway** **was activated in HAGC.** **(A, B)** GO analysis with differentially expressed genes in NAGC vs normal as well as HAGC vs normal. **(C)** Pathway specifically enriched in HAGC by GO analysis. **(D)** Heatmap representing differential expressed genes (fold change > 2) which were transcriptional targets of RelA in HAGC and NAGC.


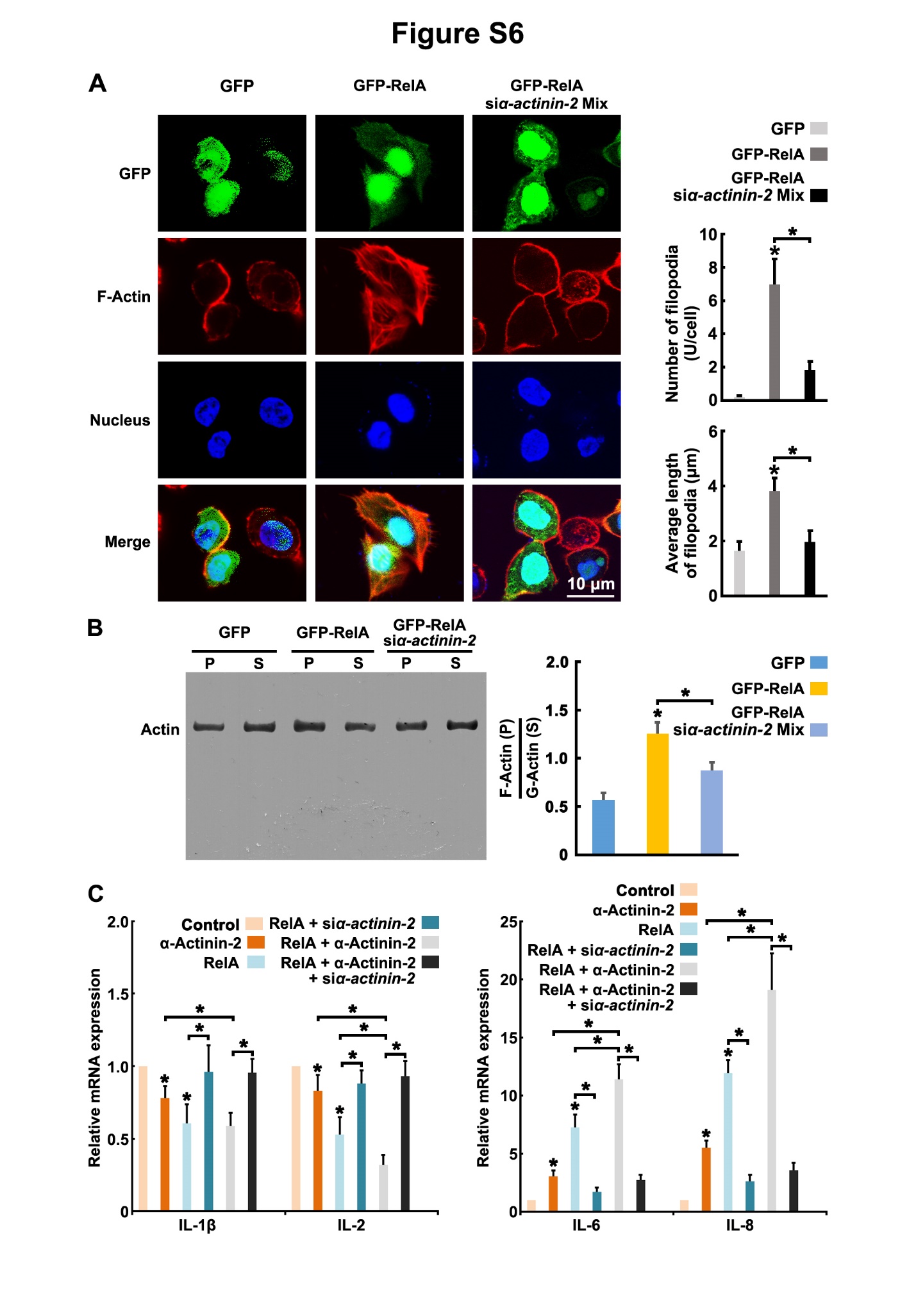


**Fig. S6 (A)** AGS cells were transfected with GFP-RelA for 2 days following transfect with negative control (NC) siRNAs or α-Actinin-2 siRNAs for 24 h, then cells were stained for quantification of the number of filopodia per cell or the average length of filopodia. GFP/GFP-α-Actinin-2 (green), F-Actin (red) and Nucleus (DAPI, blue). Scale bar, 10 μm. (**B**) GFP-RelA was co-transfected with or without α-Actinin-2 siRNAs in AGS cells for 24 h, then the ratio of F-Actin/G-Actin was determined by G-Actin and F-Actin ratio assays. P: pellet; S: supernatant. (C) AGS cells were transfected with RelA, α-Actinin-2 or RelA plus α-Actinin-2 for 2 days following transfect with negative control (NC) siRNAs or α-Actinin-2 siRNAs for 24 h, then the total RNA of cell were extracted for qRT-PCR analysis. The bars indicate the SD. The results are expressed as the mean ± SD of three independent experiments. **P* < 0.05 using Ordinary one-way ANOVA for (A and B). **P* < 0.05 using Student’s t-test for (C).

Table S1

**Clinical characteristics of patients with HAGC and NAGC**

| **Characteristics** | **HAGC % (n= 4)** | **NAGC % (n= 5)** | ***P*** |
| --- | --- | --- | --- |
| **Age (years)** |  |  |  |
| < 60 | 3 (75.0) | 3 (60.0) | 0.635 |
| ≥ 60 | 1 (25.0) | 2 (40.0) |  |
| **Sex** |  |  |  |
| Male | 3 (75.0) | 1 (20.0) | 0.099 |
| Female | 1 (25.0) | 4 (80.0) |  |
| **ECOG PS** |  |  |  |
| 0/ 1 | 0 (0.0) | 5 (100.0) | 0.003 |
| ≥ 2 | 4 (100.0) | 0 (0.0) |  |
| **Primary tumor site** |  |  |  |
| Stomach | 3 (75.0) | 4 (80.0) | 0.858 |
| GEJ | 1 (25.0) | 1 (20.0) |  |
| **Grading*** |  |  |  |
| G2 | 0 (0.0) | 3 (60.0) | 0.136 |
| G3 | 4 (100.0) | 2 (40.0) |  |

Chi-square test or Fisher’s exact test were used in Table, bold indicates *P* < 0.05.

*ECOG PS*, Eastern Cooperative Oncology Group Performance Status.

*Grading according to WHO: G1 (Well differentiated), G2 (Moderately differentiated), G3 (Poorly differentiated or undifferentiated).

Table S2

**Up-regulated genes in HAGC and/or NAGC**

| **Cancer type** | **Gene name** |
| --- | --- |
| DEGs of HAGC | FEZF1, LILRA4, **ADIPOQ**, APOE, ALPPL2, LUZP2, **FGL1**, FOXS1, THBS2, CFTR, OLFML2B, **SPINK6**, **ACTN2**, PGA3, ONECUT2, **SYT12**, |
| DEGs of NAGC | HOXA13, TFAP2A, HOXB9, HOXC6 |
| NAGC and HAGC | INHBA, HOXC9, HOXC10, HOXC11, HOXC8, ABCA13, ESM1 |

| DEGs of HAGC | Stomach cancer data in the Human Protein Atlas | | |
| --- | --- | --- | --- |
|  | ^1^Average FPKM of RNA-seq data | ^2^Protein expression in stomach cancer tissue | RNA-seq number of samples |
|  |  |  |  |
| FEZF1 | 1.2 | Not detected | 354 |
| LILRA4 | 0.3 | High/Low | 354 |
| **ADIPOQ** | **0.4** | **Not detected** | 354 |
| APOE | 139.3 | Low | 354 |
| ALPPL2 | 11 | High/Medium/Low | 354 |
| LUZP2 | 0.1 | Low | 354 |
| **FGL1** | **2.6** | **Pending for analysis** | 354 |
| FOXS1 | 4.4 | Medium/Low | 354 |
| THBS2 | 28.1 | Medium/Low | 354 |
| CFTR | 6.9 | Not detected | 354 |
| OLFML2B | 14.2 | Not detected | 354 |
| **SPINK6** | **0.1** | **Not detected** | 354 |
| **ACTN2** | **0.5** | **Not detected** | 354 |
| PGA3 | 24.8 | Medium | 354 |
| ONECUT2 | 5.4 | Not detected | 354 |
| **SYT12** | **0.4** | **Not detected** | 354 |
| **Threshold:** average FPKM < 1 and no protein was detected in stomach cancer tissues **Notes:**  1. RNA-seq data is generated by The Cancer Genome Atlas.  2. Samples used for immunohistochemistry by the Human Protein Atlas do not correspond to samples in The Cancer Genome Atlas dataset.  **DEGs:** differentially expressed genes **FPKM:** number Fragments Per Kilobase of exon per Million reads. | | | |
|  |  |  |  |
|  |  |  |  |

Table S3

**Plasmids and qRT-PCR primers**

| **Plasmids** | |
| --- | --- |
| pSIN-GFP-ADIPOQ | F:  GGACGAGCTGTACAAGGCTAGCATGATGCTGTTGCTGGGAGCTGTTC |
|  | R: TGCGGATCACTAGTGCTAGCTCAGTTGGTGTCATGGTAGAGAAGAAAGC |
| pSIN-GFP- FGL1 | F: GGACGAGCTGTACAAGGCTAGCATGATGGCAAAGGTGTTCAGTTTCATCC |
|  | R: TGCGGATCACTAGTGCTAGCTTAAATTACATTTGGAATAAAATCATTTGGCC |
| pSIN-GFP- SPINK6 | F:  GGACGAGCTGTACAAGGCTAGCATG ATGAAACTGTCAGGCATGTTTCTGC |
|  | R:  TGCGGATCACTAGTGCTAGCTCAGCATTTTCCAGGATGCTTTAGGC |
| pSIN-GFP- ACTN2 | F:  GGACGAGCTGTACAAGGCTAGCATG ATGAACCAGATAGAGCCCGGCG |
|  | R:  TGCGGATCACTAGTGCTAGCTCACAGATCGCTCTCCCCGTAGA |
| pSIN-GFP- SYT12 | F:  GGACGAGCTGTACAAGGCTAGCATG ATGGCTGTGGATGTGGCAGAATAC |
|  | R:  TGCGGATCACTAGTGCTAGCCTAGTTTCGCCGGACAGCGTG |
| pluciferease-ACTN2 | F:  gctagcgaattcGCCACCATGAACCAGATAGAGCCCGGC |
|  | R:  cgtcgtccttgtagtcggatccCAGATCGCTCTCCCCGTAGAG |
| **qRT-PCR primers** | |
| ADIPOQ | F: AACATGCCCATTCGCTTTACC |
|  | R: TAGGCAAAGTAGTACAGCCCA |
| FGL1 | F: ATGGCAAAGGTGTTCAGTTTCA |
|  | R: ACAATCTGCATACTGCCTCTTG |
| SPINK6 | F: TGACTGTGGTGAGTTCCAGGA |
|  | R: CCACTTTTCACTATGGCCTTACA |
| ACTN2 | F: CAAACCTGACCGGGGAAAAAT |
|  | R: CTGAATAGCAAAGCGAAGGATGA |
| SYT12 | F: CAGAATACCATCTGAGCGTCATC |
|  | R: TAGTCGTAATTGGGGAACGGA |
| IL-1β | F: CAACAGGCTGCTCTGGGATT |
|  | R: CCATCATTTCACTGGCGAGC |
| IL-2 | F: AACCTCAACTCCTGCCACAA |
|  | R: GCATCCTGGTGAGTTTGGGA |
| IL-6 | F: AGCCCACCGGGAACGAAAG |
|  | R: CCGAAGGCGCTTGTGGAG |
| IL-8 | F: CTCCAAACCTTTCCACCCCA |
|  | R: TTCTCAGCCCTCTTCAAAAACT |

Table S4

**Clinical characteristics of patients detected by multiple immunofluorescence staining of α-Actinin-2 and p-RelA**

| **Characteristics** | **HAGC % (n= 11)** | **NAGC % (n= 32)** | ***P*** |
| --- | --- | --- | --- |
| **Age (years)** |  |  |  |
| < 60 | 7 (63.6) | 19 (59.4) | 0.803 |
| ≥ 60 | 4 (36.4) | 13 (40.6) |  |
| **Sex** |  |  |  |
| Male | 6 (54.5) | 6 (18.8) | **0.022** |
| Female | 5 (45.5) | 26 (83.1) |  |
| **ECOG PS** |  |  |  |
| 0/ 1 | 1 (9.1) | 27 (84.4) | **< 0.0001** |
| ≥ 2 | 10 (90.9) | 5 (15.6) |  |
| **Primary tumor site** |  |  |  |
| Stomach | 8 (72.7) | 28 (87.5) | 0.252 |
| GEJ | 3 (27.3) | 4 (12.5) |  |
| **Grading*** |  |  |  |
| G1 | 3 (27.3) | 2 (6.3) | 0.100 |
| G2 | 2 (18.2) | 3 (9.4) |  |
| G3 | 6 (54.5) | 27 (84.4) |  |

Chi-square test or Fisher’s exact test were used in Table, bold indicates *P* < 0.05.

*ECOG PS*, Eastern Cooperative Oncology Group Performance Status.

*Grading according to WHO: G1 (Well differentiated), G2 (Moderately differentiated), G3 (Poorly differentiated or undifferentiated).
